# Fully Automatable Relative Binding Free Energy Calculations with Enhanced Sampling Using FAST/MBAR

**DOI:** 10.1021/acs.jctc.6c00963

**Published:** 2026-07-23

**Authors:** Miroslav Suruzhon, Justina Ratkeviciute, Khaled Abdel-Maksoud, Anna Cavalleri, Michael S. Bodnarchuk, Antonella Ciancetta, Ian D. Wall, Jonathan W. Essex

**Affiliations:** † School of Chemistry and Chemical Engineering, 7423 University of Southampton, Highfield, Southampton SO17 1BJ, U.K.; ‡ Computational Chemistry, R&D Oncology, AstraZeneca, Cambridge CB4 0WG, U.K.; § Sygnature Discovery Limited, BioCity, Pennyfoot St, Nottingham NG1 1GR, U.K.; ∥ Department of Chemical, Pharmaceutical and Agricultural Sciences (DOCPAS), University of Ferrara, Via Fossato di Mortara 17/19, 44121 Ferrara, Italy; ⊥ 468087 GSK Medicines Research Centre, Gunnels Wood Road, Stevenage SG1 2NY, U.K.

## Abstract

Alchemical free energy (AFE) calculations are a useful tool in computational drug discovery. However, they typically involve relatively short (<10 ns) simulations, meaning that the initial coordinates and, more generally, the setup of the system have a significant effect on the obtained free energy values. To remedy this, we recently developed a fully adaptive version of the simulated tempering algorithm (FAST) and applied it in the context of sampling. In this work, we extend FAST to AFE calculations with and without enhanced sampling of a particular degree of freedom of interest (FAST/MBAR). We show that enhanced sampling significantly increases the mobility of the targeted degree of freedom at the cost of reduced sampling efficiency over λ space. On the other hand, the free energy calculations without explicit targeting of certain degrees of freedom retain initial-coordinate bias over longer time scales. Despite this, both protocols readily explore nanosecond-time-scale events, such as torsional rotation, due to the single-trajectory nature of FAST, making them less sensitive to the system preparation. It is shown that the robust automated nature of FAST/MBAR makes it a competitive alternative to conventional AFE methods.

## Graphical abstract

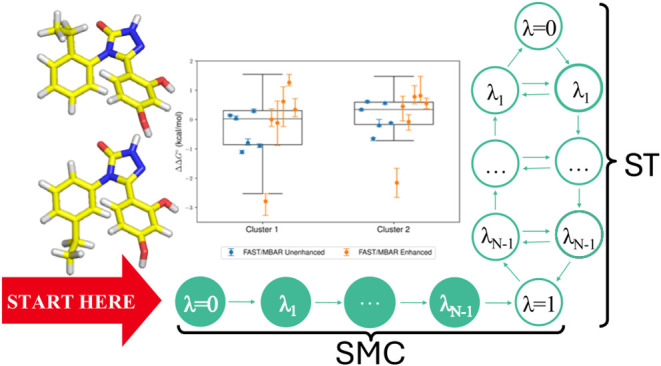

## 1 Introduction

AFE (alchemical free energy) calculations have had an increasingly significant role in computational drug discovery, prompted by the increase in GPU (graphics processing unit) power in the past decade.^1–3^ Nevertheless, their high-throughput application remains somewhat hindered by their inconsistent performance across different targets.^3^ This is not only caused by inadequate force fields, but also by insufficient sampling, suboptimal system preparation, or inefficient simulation protocols.^4^

Recent literature has demonstrated that system preparation is of central importance in MD (molecular dynamics) simulations in general and AFE calculations in particular.^1,4–9^ For example, an “incorrect” initial conformation, protonation state or binding site water molecule can all significantly affect the resulting binding free energy values quantitatively, as well as qualitatively.^5,6,10–17^ In addition, RBFE calculations require alchemical perturbation protocols that are difficult to generalize to all systems. Unless these are adaptively determined,^18–20^ one either has to risk unconverged results or incur consistently high computational costs when applying RBFE calculations on different systems.

Much of the recent literature has been concerned with proposing remedies to the above problems,^21^ including enhanced sampling approaches using parallel simulations, such as variations of REMD (replica exchange molecular dynamics).^22–27^ While these have proven themselves to be useful and robust over the years, parallel enhanced sampling methods have the disadvantage of sacrificing long-time scale sampling for the sampling of a particular degree of freedom, such as temperature, or an alchemical decoupling variable. This is problematic, since, as has been shown in recent publications, the sampling time of a typical RBFE calculation is not sufficient to explore even relatively short-time scale motions, such as dihedral rotations.^6,14,28^ This makes system preparation decisive in determining the quality of the obtained free energy result. If we are to develop robust and general free energy protocols, this effect needs to be minimized.

An enhanced sampling method which can be used both for exploring a particular degree of freedom and for performing an alchemical free energy calculation, while investing all computational time into a single simulation, is ST (simulated tempering),^29,30^ which is the serial counterpart of REMD. ST has been historically underused in comparison to REMD,^31^ largely due to its requirement for free energy estimates to achieve good sampling and higher sensitivity to kinetic barriers in temperature/parameter space. However, it is a method that has been successfully applied in free energy calculations.^32–34^ Some of its better-known variations, such as SAMS (self-adjusted mixture sampling)^35^ and TSS (Times Square sampling),^36^ address some of the limitations of ST by estimating the free energies on-the-fly and using these to adapt the sampling bias^35^ or to adaptively allocate sampling effort to undersampled alchemical intermediate states.^36^ While such approaches may improve sampling over a fixed perturbation parameter protocol, they can still be sensitive to high kinetic barriers if the perturbation protocol itself is poorly designed. In a recent publication,^37^ we extended the ST framework by adding another layer of adaptation, where the perturbation parameter protocol is also adaptively determined by the algorithm, resulting in the FAST (fully adaptive simulated tempering) method. In the context of AFE calculations, this means that more difficult alchemical perturbations will automatically be allocated more alchemical intermediates without any user input.

The fully adaptive framework presented in ref 37 has been described in the context of sampling over several distributions, where one is typically interested in only one reference distribution, with the other distributions providing increased mobility of the degrees of freedom of interest. However, FAST is a very general procedure which can be readily applied in a broader context to any ergodic discrete Markov chain of states.

In this work, we will show how this framework generalizes to the context of relative binding free energy calculations. Afterward, these calculations will be combined with the enhanced sampling protocol presented in ref 37, resulting in a hybrid algorithm: FAST/MBAR. This will allow us to not only explicitly address the sampling of certain degrees of freedom during free energy calculations, but also to minimize the correlation of the resulting free energy difference to the initial crystal structure. To do this, we will start with several preliminary theoretical considerations which extend the methodology presented in ref 37 before applying the FAST/MBAR algorithm on test cases of interest.

## 2 Theoretical Background

### 2.1 Fully Adaptive Simulated Tempering (FAST)

FAST, like ST, draws samples from a mixture distribution over the coordinates π_mix_(*x⃗*) which is a weighted sum over *N* underlying probability distributions π_*i*_(*x⃗*):

1 $$\pi_{\mathrm{mix}}\left(\mathrm{x⃗}\right)=\sum_{i=1}^{N}w_{i}\pi \left({\mathrm{\lambda ⃗}}_{i},\mathrm{x⃗}\right)$$

where $\vec{\lambda_{i}\;}$ is a vector of parameters (which may or may not be temperatures), *w*_*i*_ is the corresponding weight of the *i*-th distribution, and π­(λ⃗_*i*_, *x⃗*) is the distribution of interest. Here and henceforth, all weights will be equal to unity, which means that the relative normalization constants of the underlying distributions need to be estimated on the fly. In our implementation of FAST,^37^ this is done by using a bootstrapped form of MBAR (multistate Bennett acceptance ratio).^38^ In addition, the mixture distribution is sampled in an irreversible fashion, using a fictitious degree of freedom which specifies the direction of sampling (“lifting coordinate”).^39^

The main distinction between FAST and other implementations of ST is its protocol optimization routine, where the transition matrix **T̂**(λ⃗) is estimated using an MBAR model and its round-trip time is minimized against the protocol λ⃗ using both continuous (the values of λ⃗) and discrete (the dimension of λ⃗) optimizations. The only assumption in this procedure is that all samples are independent. Even though this assumption is not true in general, the procedure has still been shown to be applicable to a range of systems.^37^

### 2.2 Constructing the Markov Chain

The FAST method, described in Section 2.1, can be regarded as an automated procedure which automatically determines an interpolative mapping between two user-specified fixed states. In the context of enhanced sampling, these two states were defined to represent the Hamiltonian of interest and a Hamiltonian where a subset of the interactions is completely decoupled. However, these states could also correspond to two different ligands, as in the context of relative binding free energy calculations. Furthermore, this framework can be readily generalized to the case of *N* states of interest connected in an arbitrary way, the only requirement being that there are no disconnected “islands” of states, i.e., the Markov chain is ergodic (Figure 1).

**Figure 1:**
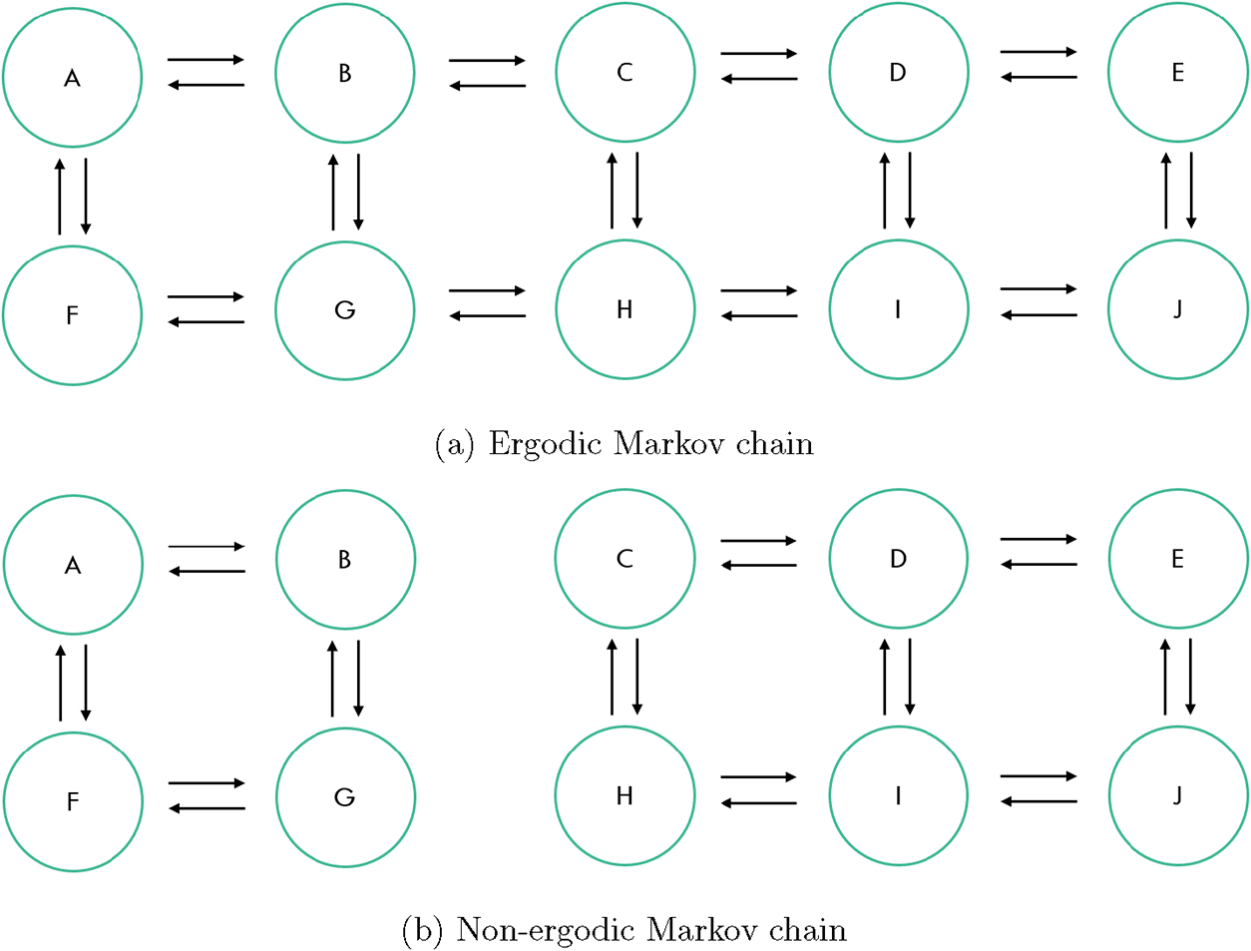
An example of an ergodic/connected (a) and a nonergodic/disconnected (b) Markov chain.

There are multiple ways to define the fixed states and their connectivity in a suitable manner for a relative binding free energy calculation with enhanced sampling (Figure 2). The simplest way to achieve this is by adding a single reference decoupled state and connecting it to one of the ligands (Figure 2a). However, one could conceivably add another reference state and connect it to the other ligand as well (Figure 2b). Another scenario arises when both of these reference states have the same Hamiltonian, in which case the corresponding Markov chain can form a closed loop (Figure 2c). Finally, one can partition the enhanced sampling of many degrees of freedom into several stages of increasing decoupling (Figure 2d).

**Figure 2:**
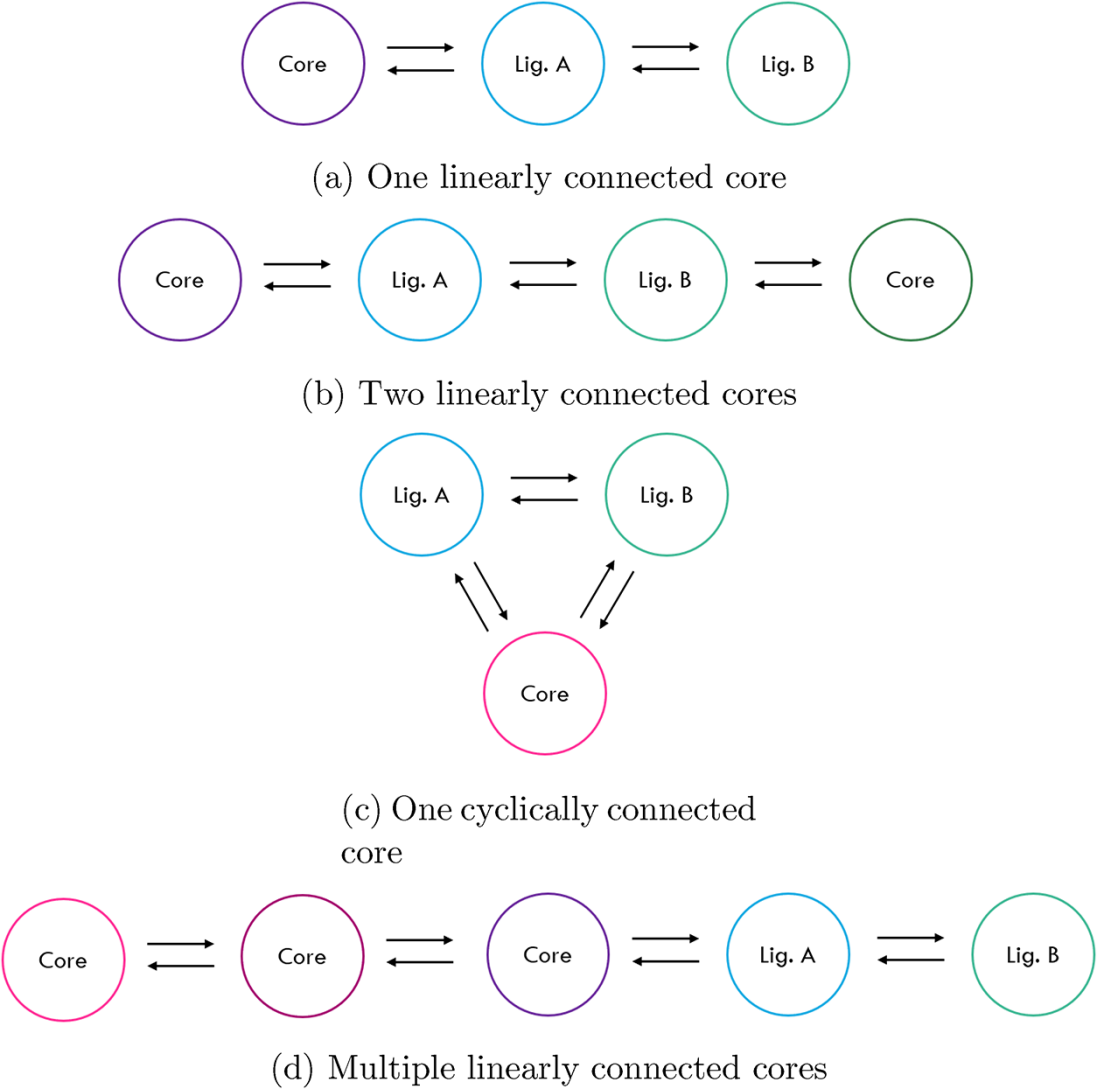
Examples of different Markov chains combining enhanced sampling and ligand–ligand perturbations.

In this work, we opt for the simplest type of Markov chain, where a single connection is made between the decoupled state and the ligand containing dummy atoms, while the two perturbed ligands will be connected in a single-topology fashion (Figure 2a). We will not consider cases where both ligands have dummy atoms, as these can always be decomposed into two separate alchemical perturbations. In this way, if the simulation starts at the decoupled state, the phase space volume relevant to the next states is always strictly decreasing, since there are more configurational states accessible to the noninteracting dummy atoms than to the fully coupled ones. This is especially relevant for the initial adaptive alchemical sequential Monte Carlo (AASMC) procedure, where it is important to minimize the sample diversity loss as the simulation progresses. Moreover, this type of Markov chain minimizes the number of state connections, thereby minimizing the computational effort dedicated to adaptation and energy evaluation over all intermediate states.

Even though we only focus on a maximum of three fixed states, this framework can be conceptually applied to an arbitrarily large number of states. These could conceivably represent different alchemical regions, effective temperatures, ligands, protonation states, or hydration levels. While all of these considerations are of potential interest for future work, they will not be addressed in the following discussion.

### 2.3 Perturbing Harmonic Bonds

The FAST protocol presented in Section 2.1 only involved the perturbation of nonbonded interactions and dihedral terms. While these considerations are sufficient in the context of enhanced sampling, a single-topology relative binding free energy calculation often requires at least one change in the harmonic bond and angle terms as well. Of particular interest to us will be the harmonic bond terms *U*_*bond*_, which are of the form:

2 $$U_{\mathrm{bond}}\left(r\right)=k_{r}(r-r_{eq}{)}^{2}$$

where *r* is the bond length, *r*_eq_ is the equilibrium bond length, and *k*_*r*_ is the harmonic force constant.

In practice, oscillations about the equilibrium bond length *r*_eq_ have low amplitudes caused by stiff force constants. This results in highly localized distributions, reminiscent of their asymptotic limit, the Dirac delta distribution^40^ (corresponding to fully constrained bond lengths). While perturbing the force field parameters is still a valid procedure in this scenario, the high localization of the distributions means that a large number of intermediate states will be needed to interpolate between the two end point equilibrium bond lengths. This can be particularly problematic when small atoms are perturbed to large atoms. For example, changing a carbon-bonded hydrogen to a bromine atom involves a bond length change as large as 0.85 Å^41^ and will as such require multiple intermediate states for sufficient overlap, thereby reducing the efficiency of the FAST procedure.

Fortunately, one can take advantage of the high stiffness of *k*_*r*_, since in practice it means that the remainder of the other much weaker forces do not significantly affect the probability distribution of the bond length. This is a prime setting for using specifically tailored MC moves to transform the bond distance independently of the other degrees of freedom. Such transformations, if possible, are much more desirable, since they can provide a direct mapping between two distributions without much loss in information, analogous to configuration mapping approaches.^42^

To achieve this, we will use reversible transformations *T⃗*_*i*→*j*_(*x⃗*_*i*_) of a sample *x⃗*_*i*_ drawn from a probability distribution π­(λ_*i*_, *x⃗*) to obey the following identity with respect to another probability distribution π­(λ_*j*_, *x⃗*):

3 $$\begin{array}{l} {\mathrm{x⃗}}_{j}={\mathrm{T⃗}}_{i\to j}\left({\mathrm{x⃗}}_{i}\right)\\ {\mathrm{x⃗}}_{i}={\mathrm{T⃗}}_{j\to i}\left({\mathrm{x⃗}}_{j}\right) \end{array}$$

This condition simply means that there is a one-to-one mapping between *x⃗*_*i*_ and *x⃗*_*j*_, given by *T⃗*(·), meaning that configurations can be transferred directly between the two distributions instead of being regenerated by sampling. The subscripts will be omitted in the following discussion for conciseness.

In practice, this requirement confines us to simple linear transformations in configuration space, such as translation and rotation. For such transformations, we can impose a type of detailed balance which relates concerted moves in configuration and λ space:

4 $$\pi \left(\lambda_{i},\mathrm{x⃗}\right)p_{\mathrm{acc}}\left(\lambda_{j},\mathrm{T⃗}\left(\mathrm{x⃗}\right)|\lambda_{i},\mathrm{x⃗}\right)d\mathrm{x⃗}=\pi \left(\lambda_{j},\mathrm{T⃗}\left(\mathrm{x⃗}\right)\right)p_{\mathrm{acc}}\left(\lambda_{i},\mathrm{x⃗}|\lambda_{j},\mathrm{T⃗}\left(\mathrm{x⃗}\right)\right)d\mathrm{T⃗}\left(\mathrm{x⃗}\right)$$

where we have assumed equal proposal probabilities *p*_prop_(λ_*j*_|λ_*i*_) = *p*_prop_(λ_*i*_|λ_*j*_) for brevity. This equation is also directly applicable to the more general case of skew detailed balance, which is used in FAST.^43^ The corresponding Metropolis acceptance criterion *p*_acc_(λ_*j*_, *T*(*x⃗*)|λ_*i*_, *x⃗*) is then:

5 pacc(λj,T⃗(x⃗)|λi,x⃗)=min[1,π(λj,T⃗(x⃗))π(λi,x⃗)dT⃗(x⃗)dx⃗]=min[1,π(λj,T⃗(x⃗))π(λi,x⃗)|JT⃗(x⃗)|]

where |**J**_*T⃗*_(*x⃗*)| denotes the Jacobian determinant corresponding to the transformation *T⃗*(*x⃗*). The usefulness of eq 4 comes from the fact that the evaluation of the potentially unfavorable π­(λ_*j*_, *x⃗*) is circumvented and is instead replaced by the more favorable π­(λ_*j*_, *T⃗*(*x⃗*)). For a bond rescaling transformation, this means that the bond length is changed to a more favorable value before evaluating π­(λ_*j*_, *T⃗*(*x⃗*)) and is reverted back to the original bond length if the transition attempt is rejected.

Here and henceforth, the simplest type of bond rescaling transformation will be considered, where the bond length is scaled by a constant scaling factor *s*, which is determined by the ratio of the parameter-dependent equilibrium bond distances *r*_eq_ at λ_*i*_ and λ_*j*_:

6 $$s\equiv \frac{r_{\mathrm{eq}}\left(\lambda_{j}\right)}{r_{\mathrm{eq}}\left(\lambda_{i}\right)}$$

In this scenario, it can be shown that |**J**_*T⃗*_(*x⃗*)| is equal to *s* ^3^ (Supporting Information Section 1). In practice, one of the bond atoms will be chosen to be stationary, while the other atoms and the rest of the molecule will be moved by this transformation. While this type of scaling is obviously reversible, it is not expected to be useful in the case of ring bond perturbations, since one cannot change the bond length without affecting the other internal degrees of freedom, and thus the other energy terms. Therefore, this procedure will only be used for rescaling bonds outside of rings, while any other degrees of freedom will not be changed.

## 3 Methods

### 3.1 System Preparation and Simulation

All system preparation and simulation methods are identical to those described in ref 37, where only the split protocol utilizing the CSC (classical soft-core potential)^44^ potential was used for all simulations with parameters *a* = 1, *b* = 1, *c* = 6 and α = 0.5. All simulations were performed in triplicate, each for 160 ns. In two cases (Hsp90 (heat shock protein 90) and PTP1B (protein tyrosine phosphatase 1B)), three additional repeats were carried out due to the increased sampling difficulty observed in these systems. Two different types of protocols were investigated for all of the systems: FAST with only two fixed states corresponding to the fully coupled ligands *A* and *B*, and FAST with enhanced sampling, with the corresponding Markov chain shown in Figure 2a. In the case of nonring bond perturbations, all λ transitions were facilitated with bond rescaling, as described in Section 2.3. During the bond rescaling procedure, the side of the bond connected only to alchemically modified atoms was chosen to be mobile, while the other side was kept static.

The FAST simulation procedure was the same as described in ref 37 using OpenMMSLICER 3.0.0. The main difference was that all λ values were discretized to three significant figures instead of two. The reason for this choice was the expected increased number of intermediate λ values for the alchemical changes explored in this work. In addition, harmonic restraints with force constants of 5 kcal/mol/Å^2^ each were added to all nonperturbed ligand atoms during the equilibration stage of the enhanced Hsp90 FAST/MBAR protocol in order to prevent a ligand binding mode change. These procedures were repeated for both the bound and solvated legs of the free energy calculations.

In the case of PTP1B (protein tyrosine phosphatase 1B), one of the ligand perturbations investigated in Suruzhon et al.^6^ was used for FAST/MBAR validation. In this case, alongside six simulations initiated from unequilibrated structures, an additional set of sextuplicate FAST/MBAR simulations was performed, where each starting structure was taken from the 20 ns equilibrated structures reported in Suruzhon et al.^6^

To compare FAST/MBAR to regular methods, triplicate AFE (alchemical free energy) calculations were run for each system in both legs in GROMACS 2018.4 using the same procedure described in ref 45. Briefly, a split alchemical protocol was used, in which the soft-core sterics and the bonded interactions were perturbed over the initial 30 λ windows, followed by the electrostatics over the subsequent 10 windows. Each window involved a 25,000-step steepest descent minimization, 50 ps NVT equilibration, and 50 ps NPT equilibration before a 4 ns NPT production. The alchemical free energy values corresponding to the PTP1B system with and without equilibration were directly taken from Suruzhon et al.^6^

### 3.2 Topology

All alchemical transformations were performed in a single-topology fashion. The implementation of these topologies in OpenMM used code from Perses,^46,47^ subsequently modified for the purposes of this study and included in OpenMMSLICER. In the implementation used hereafter, five different types of alchemical variables λ are used, each associated with a particular type of interaction: λ_bonds_, λ_angles_, λ_dihedrals_, λ_sterics_, and λ_electrostatics_. In all cases, the force field parameters (force constants, equilibrium distances and charges) are linearly interpolated between the two end values as a function of the relevant alchemical variable. The only exceptions are the dihedral terms, where their corresponding energies, rather than the associated parameters, are linearly interpolated. Finally, the nonbonded 1–4 interactions (LJ (Lennard-Jones) parameters and charge products) are also interpolated in a linear fashion. All dummy atoms assume the equilibrium bonded values of their interacting counterparts, meaning that only the force constants and charges/charge products are scaled in these cases. They are then introduced into the system using a nonbonded soft-core potential with the same functional form as described in Pham and Shirts.^44^

In the following simulations, λ_bonds_, λ_angles_, λ_dihedrals_, and λ_sterics_ were always changed simultaneously and independently of λ_electrostatics_ (split protocol). However, a generalized alchemical variable λ will be instead reported in the text for brevity, following the convention in ref 37. For the three-state FAST/MBAR protocols, λ corresponds to the common core at λ = 0 and to the fully coupled ligands *A* and *B* at λ = 0.5 and λ = 1 respectively. Any interpolation between these states is then performed identically to the two-state protocols.

### 3.3 Analysis

All reported time-dependent free energy distributions were obtained after 10-fold bootstrapping of the decorrelated samples as described in ref 37. The effective decorrelation time τ_decorr_ used for removing correlated samples was obtained from its final estimate during the FAST/MBAR run, where it is calculated by comparing the observed round trip time, defined as the time taken to transition from λ = 0 to λ = 1 and back, with that predicted from the MBAR-derived transition matrix. This procedure was repeated for both the bound and solvated legs. The median of all final bootstrapped free energy values over all replicates of the enhanced solvated leg FAST/MBAR protocol was then consistently subtracted from all bound legs for this system in order to report relative binding free energy values. Since in all cases the solvated legs displayed an order of magnitude lower variability than the bound legs, all of the observed variance has been attributed to the much more practically interesting bound legs.

To assess the effectiveness of the protocol optimization, the number of round trips per nanosecond was also reported, together with the ratio of samples obtained at λ = 1 relative to λ = 0, $\frac{N_{1}}{N_{0}}$ , which measures the balance of sampling between the two end states. The effective decorrelation time, as well as the round trip rate, were reported as their arithmetic averages and standard deviations, while the geometric mean and standard deviation were used for $\frac{N_{1}}{N_{0}}$ to better represent variability when the number of samples at one end state greatly exceeds that at the other.

To determine the dependence of the calculated *Δ*Δ*G* ^⊖^ values on different ligand conformers, dihedral clustering was performed on the marginal distributions of each relevant ligand torsion at all λ values. All cluster boundaries were defined manually before extracting only the relevant simulation frames belonging to a particular cluster. If more than one relevant conformation was observed, these were then used to obtain bootstrapped cluster-dependent free energies as described in the previous paragraph. The kinetics and the slowest implied time scales obtained from Markov state models (MSMs) were determined using the discretized clusters to fit a Bayesian hidden MSM,^48,49^ as implemented in PyEMMA 2.5.12.^50^ The estimator fitting procedure was performed using the default settings to generate 100 different transition matrices over a range of different lag times τ in the range of 5–100 ps. The distributions of the slowest implied time scales *t*_slowest_(τ) from each of these lag times were subsequently obtained using the formula:^51^

7 $$t_{\mathrm{slowest}}\left(\tau \right)=-\frac{\tau }{\ln\lambda_{\mathrm{slowest}}\left(\tau \right)}$$

where λ_slowest_ is the second largest eigenvalue of the corresponding stochastic matrix. In almost all cases, the lag time for calculating the mean and sample standard deviation of *t*_slowest_(τ) was chosen to be 50 ps. The only exception was the kinetic analysis corresponding to the ligand ethyl group rotation in Hsp90, where a lag time of 20 ps was instead used due to the fast substituent motion and poor MSM accuracy at longer lag times.

In two cases (Hsp90 and PTP1B) clustering of the ligand common core was performed to investigate the dynamics of some unenhanced rare events over time. The method used to perform this clustering followed the procedure used on the TGF-β (transforming growth factor β) ligand common core described in ref 37. The atoms used to obtain the center of geometry were chosen to be the six dihydroxybenzene ring atoms (Hsp90) and the five thiophene ring atoms (PTP1B).

Where relevant, the Kruskal–Wallis rank-based test of statistical significance was used to compare several groups of *Δ*Δ*G* ^⊖^ values as well as efficiency metrics. This test verified the null hypothesis that the mean ranks of the samples drawn from each of the groups are the same, although it should be noted that a rejected null hypothesis may reflect differences in sample dispersions rather than medians alone. The resulting *p*-values obtained using SciPy^52^ were accordingly reported. To estimate uncertainty for reported differences between groups, confidence intervals were estimated by bootstrap resampling of the corresponding values in each group using SciPy, and reported as 95% CI.

As the main focus of this work is internal method consistency, comparison to available experimental values is provided in the Supporting Information (Section 2).

## 4 Results

### 4.1 Coagulation Factor Xa (FXa)

The first test case for validating FAST/MBAR is FXa (coagulation factor Xa) bound to a medium-sized ligand (PDB ID: 1LQD^53^), where a fluoride group is perturbed to a methyl group (Figure 3b,c). As previously described, two FAST/MBAR protocols are compared: unenhanced FAST with only the two fixed end point states, and FAST with enhanced sampling of selected ligand torsions. In the enhanced FAST/MBAR protocol, the two torsions closest to the perturbed group, as well as an additional distal hydroxide torsion, were explicitly decoupled (Figure 3a). The latter torsion was added purely for demonstrative purposes, as it is expected to be straightforward to sample with alchemical methods without much performance penalty.

**Figure 3:**
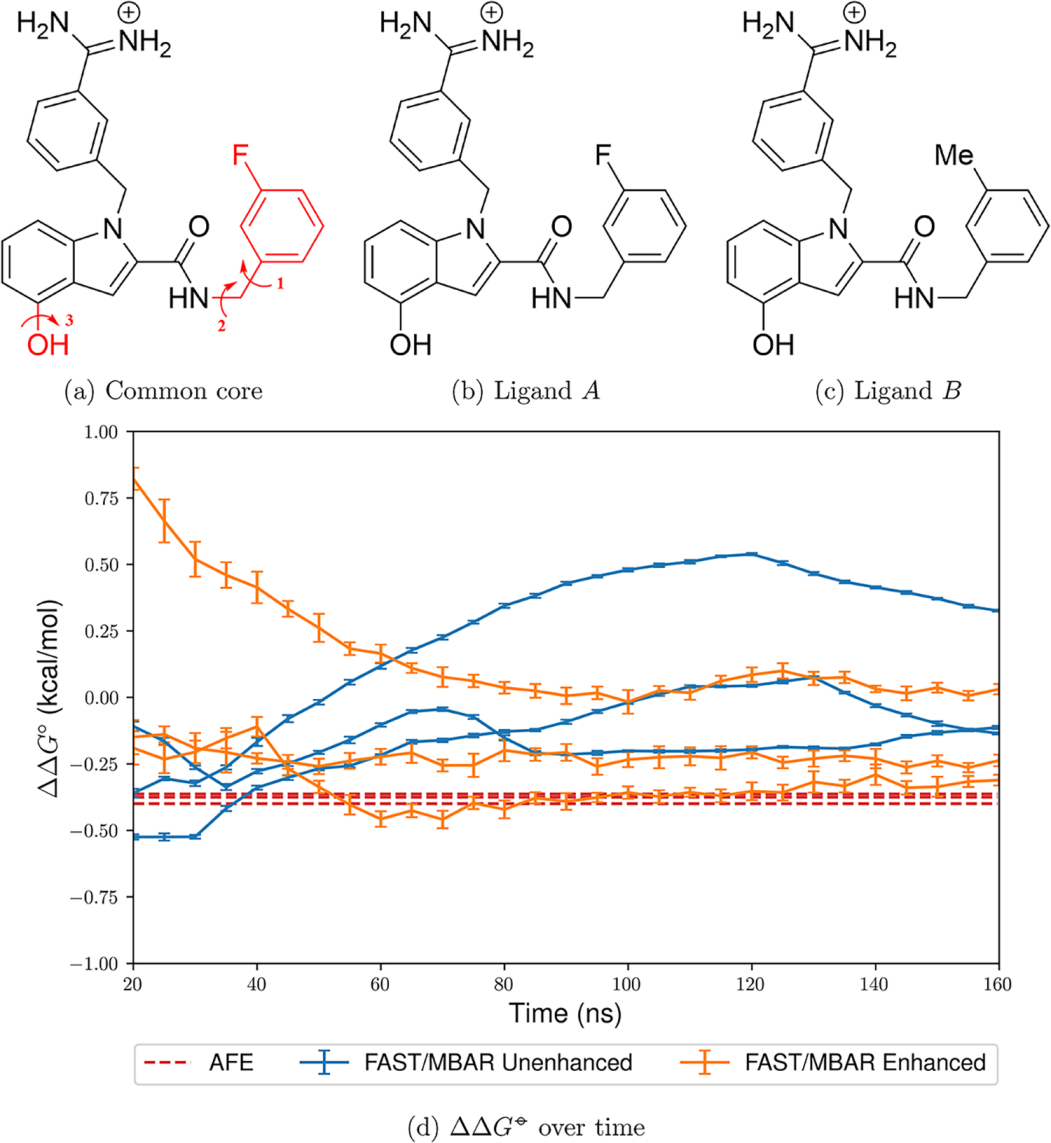
Common core of the FXa ligand with dummy atoms and bonds in red (a), the two ligands constituting the relative free energy perturbation (b and c) and the bootstrapped *Δ*Δ*G* ^⊖^ estimates corresponding to both FAST/MBAR protocols over time (d). In (d), the median *Δ*Δ*G* ^⊖^ for each replicate is plotted over time with associated error bars determined by the bootstrapped MAD, while *Δ*Δ*G* ^⊖^ determined by each AFE replicate is shown as a separate red line.

The behavior of the FAST/MBAR protocol with and without enhanced sampling shows a time-dependent trend (Figure 3d). At short time scales (20 ns), the difference between the median free energy values obtained from the two protocols is 0.21 kcal/mol (95% CI: −0.08 to 1.35 kcal/mol; Kruskal–Wallis *p* = 0.28), while at longer time scales (160 ns), this difference decreases to 0.06 kcal/mol (95% CI: −0.65 to 0.18 kcal/mol; Kruskal–Wallis *p* = 0.28). In addition, the values obtained by AFE calculations are comparable to the FAST/MBAR protocol without extra sampling after 20 ns, with a median difference of 0.02 kcal/mol (95% CI: −0.16 to 0.29; Kruskal–Wallis *p* = 0.51). In this way, the unenhanced FAST/MBAR protocol outputs free energy values consistent with AFE calculations, before slowly converging to the values predicted by the enhanced FAST/MBAR protocol. This suggests that the rare events explicitly sampled by the enhanced protocol are explored by the unenhanced one simply by virtue of long-time scale MD (molecular dynamics) sampling, although the overall differences between protocols remain small across all time scales.

Analysis of the obtained *Δ*Δ*G* ^⊖^ values from different ligand conformers reveals that torsion 2, shown in Figure 3a, results in the highest free energy discrepancies. Its three different conformations (Figure 4a–c) result in median free energy values of −0.07, −0.07, and −0.28 kcal/mol, respectively (Figure 4d), although these differences are not significantly different (Kruskal–Wallis *p*-value = 0.71). MSM analysis of torsion 2 reveals that explicit enhancement results in an approximately 5-fold decrease of its slowest implied time scale of the transition (from 2.44 ± 0.23 ns to 0.47 ± 0.03 ns), suggesting that this torsion is likely the main source of short-time scale discrepancies between the two protocols. While implied time scales of ∼2 ns are only marginally accessible to the AFE protocol used in this study, they can be readily explored by the unenhanced FAST/MBAR protocol over 160 ns, which is why the two FAST/MBAR protocols have a better agreement with each other at these longer time scales.

**Figure 4:**
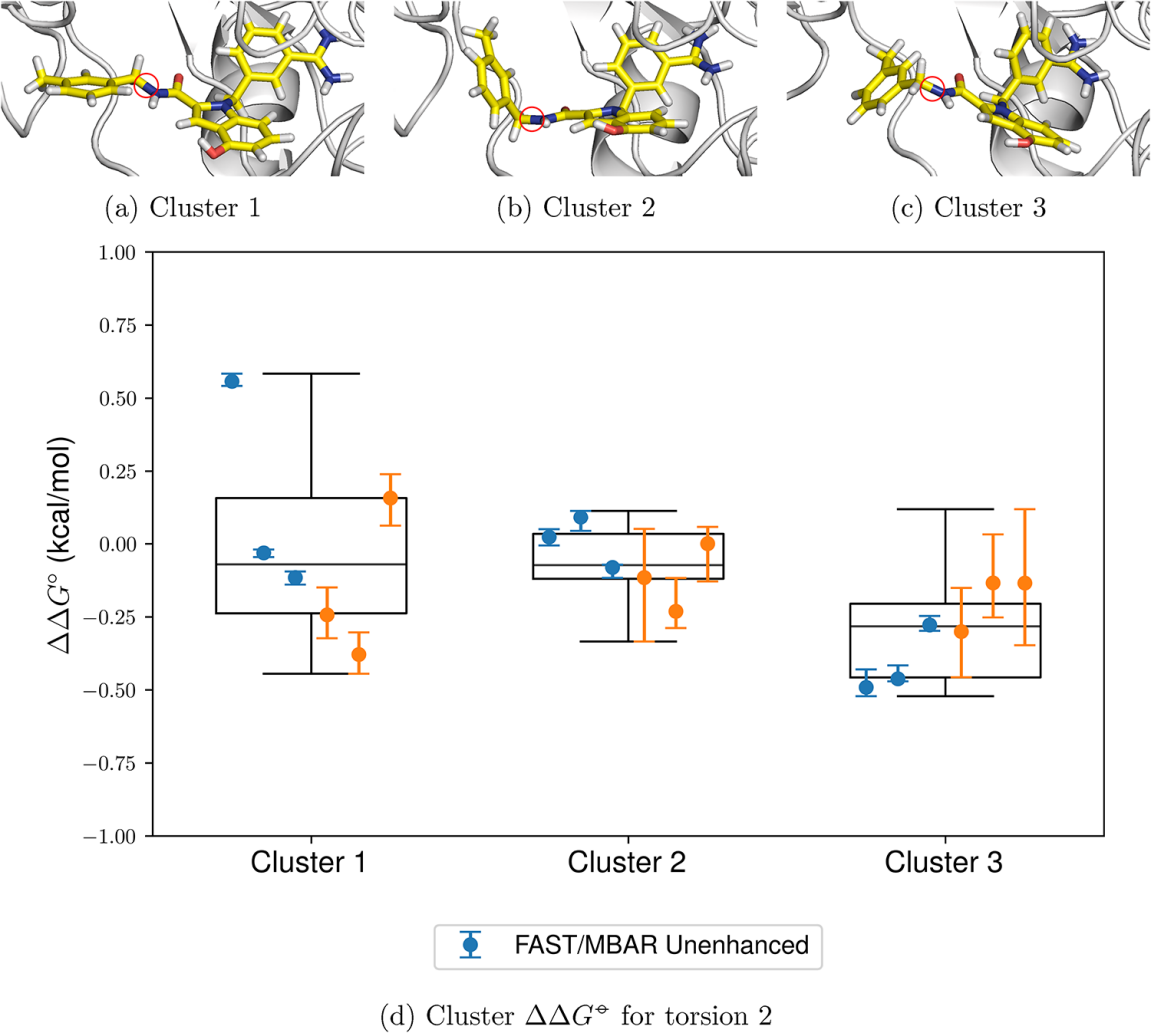
Three different clusters corresponding to torsion 2 (circled in red) in the FXa ligand (a–c) and their respective *Δ*Δ*G* ^⊖^ values obtained from both FAST/MBAR protocols (d). The gray lines represent the median of the total data set, the boxes include the interquartile range, while the whiskers extend to the 5th and 95th percentiles. Each data point represents the median bootstrapped value for each replicate, and the error bars extend between the minimum and maximum bootstrapped values.

Both FAST/MBAR protocols require a small number of λ values, with the unenhanced protocol converging to a median of only 3 final values with a standard deviation of 0, compared to 5 ± 0 values generated by the initial AASMC procedure. In contrast, the optimal enhanced FAST/MBAR protocol has 14 ± 3 λ values compared to 20 ± 1 windows generated by AASMC. Similarly to the results in,^37^ it can be seen that the adaptive protocol optimization procedure is independent of the initial AASMC protocol and generates the shortest possible λ schedules, with the perturbation of a fluoride to a methyl group only requiring one intermediate λ window. This also shows that the bond rescaling routine is extremely efficient at reducing the number of intermediates when the bond length is changed.

The efficiency of the protocols is reflected by the round-trip rates between λ = 0 and λ = 1 per nanosecond: 33.31 ± 1.89 for the unenhanced protocol and 0.33 ± 0.03 for the enhanced protocol, respectively (Table 1). Despite the latter protocol being 100 times less efficient than the former (Kruskal–Wallis *p* = 0.05), this round-trip rate is still satisfactory over the time scales studied (160 ns). While this 100-fold difference in efficiency can be largely attributed to the longer λ protocols when some of the torsions are explicitly enhanced, the effective decorrelation time τ̂_decorr_ for the enhanced protocol is still ∼5 times higher: 6.44 ± 1.04 ps compared to 1.21 ± 0.02 ps for the unenhanced (Kruskal–Wallis *p* = 0.05) (Table 2). Therefore, the extra sampling creates an additional kinetic barrier in λ space, which increases τ_decorr_. This observation is also supported by the higher deviation from unity of the ratio of samples between λ = 1 and λ = 0, $\frac{N_{1}}{N_{0}}$ , for the enhanced protocol: 3.64 ± 1.93 versus 0.78 ± 1.18 for the unenhanced (Kruskal–Wallis *p* = 0.05) (Table 3), meaning that, in this case, λ = 0 is substantially undersampled by the enhanced protocol.

**Table 1:** Number of Round Trips Per Nanosecond for Each of the Systems across Different Replicates and FAST/MBAR Protocols^a^

		Replicate						Total	
System	Enhanced	1	2	3	4	5	6	Average	St. dev.
FXa	No	32.40	35.48	32.06				33.31	1.89
	Yes	0.37	0.33	0.30				0.33	0.03
Thrombin	No	21.85	21.24	17.34				20.15	2.45
	Yes	0.18	0.18	0.16				0.17	0.01
Hsp90	No	10.58	8.89	7.22	6.62	9.44	6.75	8.25	1.63
	Yes	0.03	0.11	0.11	0.09	0.22	0.38	0.15	0.12
PTP1B w/o Equilibration	No	1.18	1.36	2.14	1.76	1.59	1.37	1.57	0.35
	Yes	0.08	0.06	0.04	0.10	0.04	0.08	0.06	0.02
PTP1B with Equilibration	No	1.34	1.90	2.09	1.24	0.74	2.26	1.60	0.58
	Yes	0.08	0.06	0.04	0.07	0.09	0.11	0.07	0.02

a The averages and the corresponding standard deviations are also given.

**Table 2:** Final Measured *τ̂*_decorr_ in ps for Each of the Systems across Different Replicates and FAST/MBAR Protocols^a^

		Replicate						Total	
System	Enhanced	1	2	3	4	5	6	Average	St. dev.
FXa	No	1.20	1.23	1.21				1.21	0.02
	Yes	5.46	6.31	7.53				6.44	1.04
Thrombin	No	1.42	1.37	1.32				1.37	0.05
	Yes	11.59	11.71	13.60				12.30	1.13
Hsp90	No	1.43	1.52	1.58	1.96	1.53	1.90	1.65	0.22
	Yes	34.08	9.09	9.78	13.68	5.60	3.04	12.54	11.17
PTP1B w/o Equilibration	No	3.47	3.29	1.86	2.28	2.73	3.28	2.82	0.64
	Yes	7.62	5.92	8.32	5.04	19.12	7.53	8.92	5.14
PTP1B with Equilibration	No	3.06	2.14	1.88	3.35	5.69	1.84	2.99	1.47
	Yes	7.31	9.64	13.01	7.89	6.86	5.45	8.36	2.66

a The averages and the corresponding standard deviations are also given.

**Table 3:** Fraction of Total Samples between λ = 1 and λ = 0, $\frac{N_{1}}{N_{0}}$ , for Each of the Systems across Different Replicates and FAST/MBAR Protocols^a^

		Replicate						Total	
System	Enhanced	1	2	3	4	5	6	Average	St. dev.
FXa	No	0.68	0.94	0.74				0.78	1.18
	Yes	2.27	2.75	7.71				3.64	1.93
Thrombin	No	2.81	2.86	1.17				2.11	1.67
	Yes	1.55	2.43	6.04				2.83	2.00
Hsp90	No	0.94	0.54	1.21	0.68	0.47	0.79	0.73	1.42
	Yes	4.03	6.71	0.20	0.47	1.05	0.80	1.13	3.74
PTP1B w/o Equilibration	No	0.25	0.86	1.20	0.57	0.68	0.55	0.62	1.70
	Yes	1.95	6.39	0.99	0.63	6.53	2.21	2.20	2.59
PTP1B with Equilibration	No	0.78	0.73	0.84	0.55	0.97	1.08	0.81	1.27
	Yes	0.34	0.26	0.25	5.56	1.01	1.47	0.75	3.42

a The geometric averages and the corresponding geometric standard deviations are also given.

### 4.2 Thrombin

Thrombin bound to a medium-sized ligand is another system which constitutes a practically relevant test case for FAST/MBAR (PDB ID: 2ZFF^54^). Let us consider the simple perturbation of a methyl group to an ethyl group, where the enhanced FAST/MBAR protocol explicitly handles the rotation of the two torsions closest to the alchemical perturbation (Figure 5a–c). In this way, we will be able to compare the quality of sampling of the tolyl ring between both FAST/MBAR protocols.

**Figure 5:**
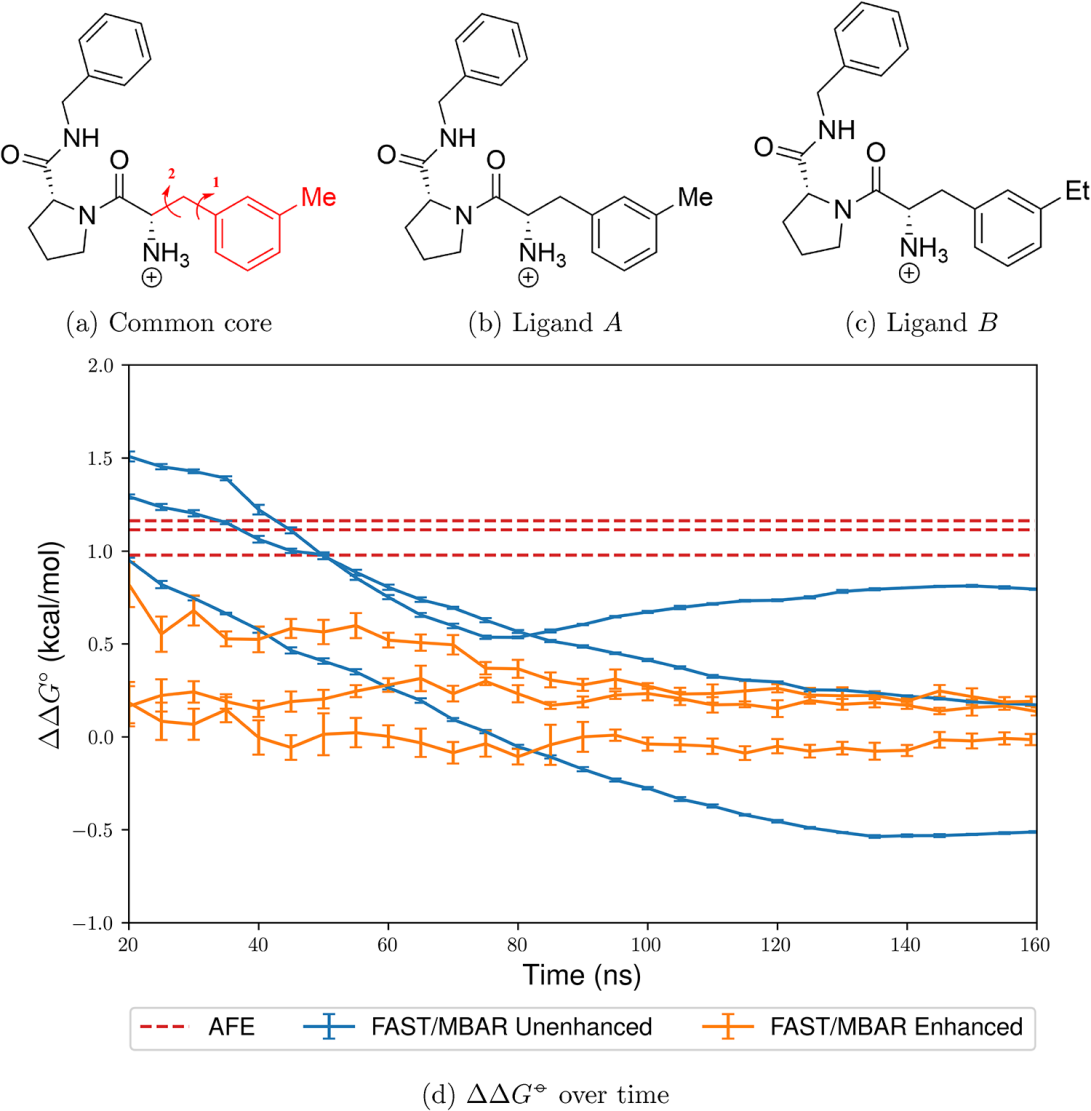
Common core of the thrombin ligand with dummy atoms and bonds in red (a), the two ligands constituting the relative free energy perturbation (b and c) and the bootstrapped *Δ*Δ*G* ^⊖^ estimates corresponding to both FAST/MBAR protocols over time (d). In (d), the median *Δ*Δ*G* ^⊖^ for each replicate is plotted over time with associated error bars determined by the bootstrapped MAD, while *Δ*Δ*G* ^⊖^ determined by each AFE replicate is shown as a separate red line.

The free energy results from both FAST/MBAR protocols closely follow the behavior observed in the FXa test case. Here, the median free energy difference between both protocols after only 20 ns of sampling is 1.11 kcal/mol (95% CI: −1.34 to −0.13 kcal/mol; Kruskal–Wallis *p* = 0.05), decreasing to 0.05 kcal/mol after 160 ns (95% CI: −0.81 to 0.70 kcal/mol; Kruskal–Wallis *p* = 0.83) (Figure 5d). Similarly, the AFE results agree well with the unenhanced FAST/MBAR protocol after 20 ns with a median difference of 0.17 kcal/mol (95% CI: −0.21 to 0.53 kcal/mol; Kruskal–Wallis *p* = 0.51). In this way, it can again be seen that FAST/MBAR at shorter time scales closely follows the AFE results before eventually approaching the free energy value obtained by the enhanced protocol. This again suggests the presence of a slow transition to a more thermodynamically relevant state which is not captured by short-time scale sampling.

The main source of these free energy discrepancies is in torsion 1 shown in Figure 5a, which has two conformations (Figure 6a,b) with associated median *Δ*Δ*G* ^⊖^ values of 0.51 and −0.19 kcal/mol, respectively (Figure 6c). This significant difference (Kruskal–Wallis *p*-value ≪ 0.001) shows that the choice of initial conformer introduces non-negligible bias and adequate sampling is required for reliable free energy calculations. Similarly to FXa, the kinetics of this torsion are substantially accelerated by explicit sampling, with the slowest implied time scale being 25.15 ± 9.48 ns and 0.39 ± 0.02 ns for the unenhanced and enhanced FAST/MBAR protocols, respectively. Despite the much higher torsional kinetic barrier compared to that observed for FXa, it is clear that this long implied time scale can still be explored by the unenhanced 160 ns FAST/MBAR protocol, even if the sampling is not as efficient as in the explicitly enhanced protocol. In this way, this test case once again demonstrates the sampling advantage of FAST/MBAR over conventional AFE calculations.

**Figure 6:**
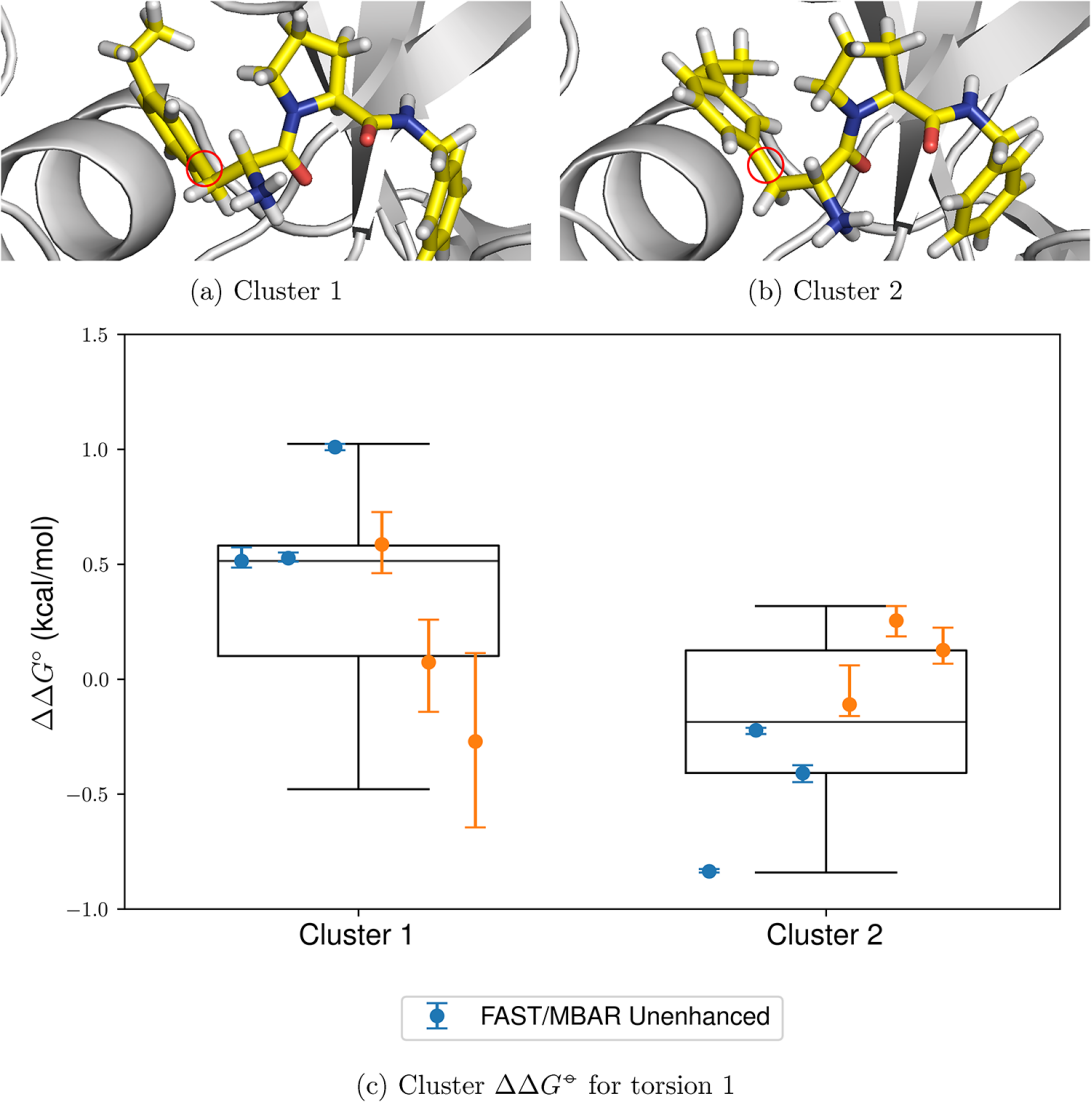
Two different clusters corresponding to torsion 1 (circled in red) in the thrombin ligand (a and b) and their respective *Δ*Δ*G* ^⊖^ values obtained from both FAST/MBAR protocols (c). The gray lines represent the median of the total data set, the boxes include the interquartile range, while the whiskers extend to the 5th and 95th percentiles. Each data point represents the median bootstrapped value for each replicate, and the error bars extend between the minimum and maximum bootstrapped values.

The protocol efficiency of both FAST/MBAR schedules is again demonstrably higher than the initial protocols output by AASMC. For FAST/MBAR without explicit sampling enhancement, the final average protocol length is 4 ± 0 λ windows, compared to 7 ± 1 initial λ values. Similarly, the enhanced FAST/MBAR protocol only needs an average of 13 ± 3 λ windows, as opposed to 19 ± 1 intermediates output by AASMC. This behavior is comparable to FXa, once again demonstrating the parsimony of the protocol optimization procedure.

As expected, the shorter protocols of the unenhanced FAST/MBAR algorithm result in high round-trip rates per nanosecond: 20.15 ± 2.45, versus 0.17 ± 0.01 for the enhanced FAST/MBAR protocol (Kruskal–Wallis *p* = 0.05) (Table 1), again showing a 2 orders of magnitude difference in efficiency. Although the average τ̂_decorr_ of the FAST/MBAR protocol without explicit sampling enhancement is only 1.37 ± 0.05 ps, the enhanced protocol exhibits a much higher effective correlation with τ̂_decorr_ of 12.30 ± 1.13 (Kruskal–Wallis *p* = 0.05) (Table 2), indicating the presence of substantial kinetic barriers in λ space introduced by the extra sampling. Unsurprisingly, the less efficient protocol also exhibits a larger $\frac{N_{1}}{N_{0}}$ with a geometric mean of 2.83 ± 2.00 compared to 2.11 ± 1.67 for the unenhanced protocol (Kruskal–Wallis *p* = 0.83) (Table 3).

### 4.3 Heat Shock Protein 90 (Hsp90))

The next test case for FAST/MBAR is Hsp90 bound to a ligand containing three rings (PDB ID: 5J64 ^55^). Here we consider the perturbation of an ethyl group to a fluoride substituent, with the enhanced FAST/MBAR protocol exploring the two torsions closest to the perturbation, as well as the simple dihedral rotations of two hydroxyl groups (Figure 7a–c). This system has been previously considered in the context of nonequilibrium free energy calculations, where it was found that rare binding site events can drastically hinder the sampling efficiency thereof.^56^

**Figure 7:**
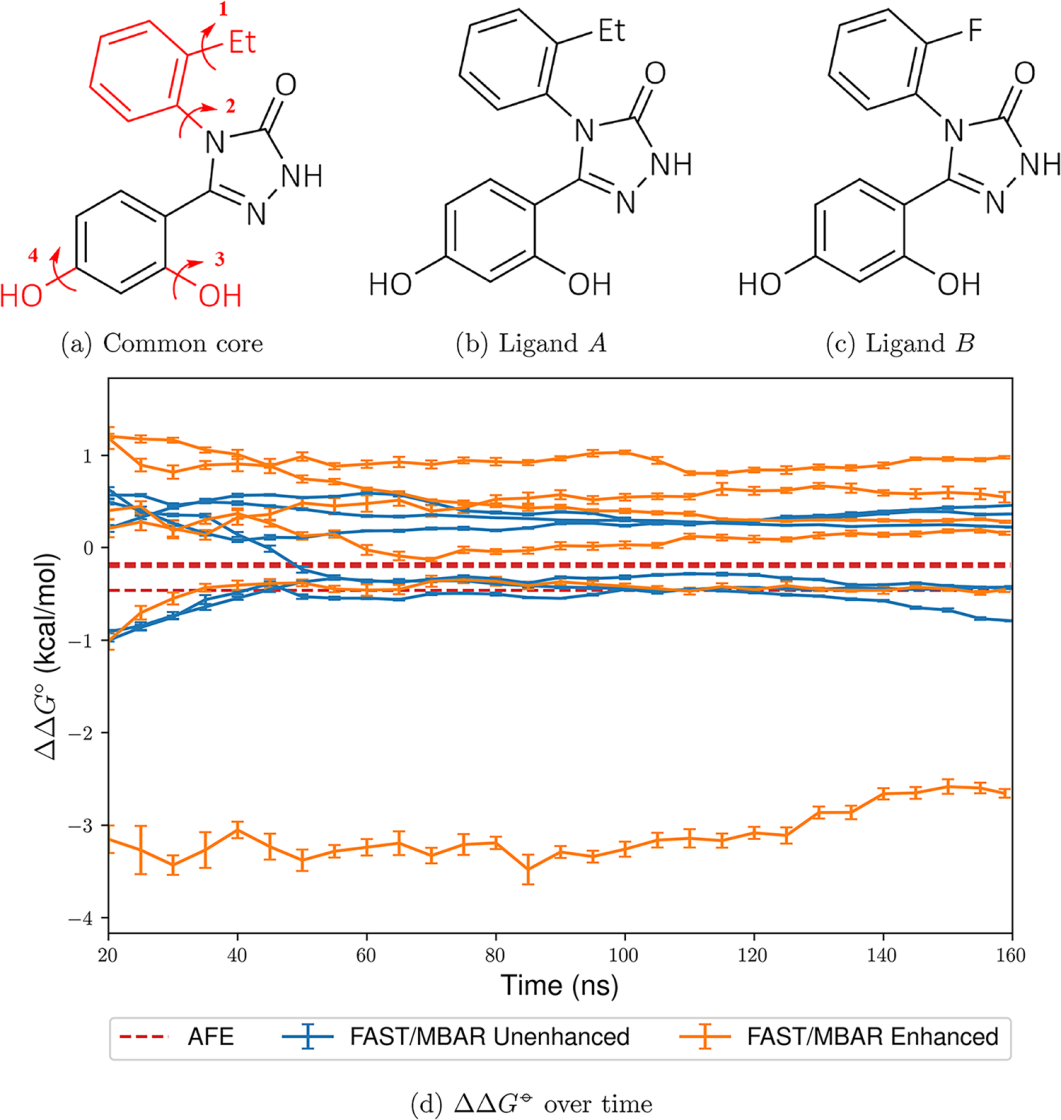
Common core of the Hsp90 ligand with dummy atoms and bonds in red (a), the two ligands constituting the relative free energy perturbation (b and c) and the bootstrapped *Δ*Δ*G* ^⊖^ estimates corresponding to both FAST/MBAR protocols over time (d). In (d), the median *Δ*Δ*G* ^⊖^ for each replicate is plotted over time with associated error bars determined by the bootstrapped MAD, while *Δ*Δ*G* ^⊖^ determined by each AFE replicate is shown as a separate red line.

In contrast to the previous test cases, the behavior of the free energy estimates of both FAST/MBAR protocols over time shows poor convergence, with a final free energy MAD (mean absolute deviation) of 0.79 and 0.45 kcal/mol for the enhanced and unenhanced protocols, respectively (Figure 7d). In this case, the median free energy deviation between both protocols is 0.04 kcal/mol after 20 ns (95% CI: −2.25 to 1.61 kcal/mol; Kruskal–Wallis *p* = 0.94), increasing to 0.32 kcal/mol after 160 ns (95% CI: −1.48 to 1.04 kcal/mol; Kruskal–Wallis *p* = 0.75). Interestingly, the free energy estimates by AFE calculations yield values closer to the unenhanced protocol after 160 ns, with median difference of 0.11 kcal/mol (95% CI: −0.42 to 0.83 kcal/mol; Kruskal–Wallis *p* = 0.61) compared to 0.56 kcal/mol after 20 ns (95% CI: −0.75 to 1.03 kcal/mol; Kruskal–Wallis *p* = 0.44)—behavior which is inconsistent with previous test cases.

The main source of the increased variability in the estimated free energies is one of the enhanced FAST/MBAR simulations, which results in a final free energy difference of −2.66 kcal/mol. Correlated with this behavior is the kinetic trapping of this simulation in λ space which deteriorates the sampling quality (Figure 8). These kinetic barriers are caused by slow transitions of the common core, resulting in two main common core conformers (Figure 8a,b). Since the second cluster is predominantly observed at lower λ values before returning to the first cluster, it can be deduced that these transitions between clusters are promoted by the increased mobility at the bottom half of the λ protocol, resulting in kinetically trapped states which create a bottleneck in the simulation. Since the states relevant for the free energy estimation (0.5 ≤ λ ≤ 1.0) are not sampled equally after ∼50 ns, it can be conjectured that the anomalous free energy behavior of this simulation is primarily caused by these rare events. These observations are consistent with those of Baumann et al.,^56^ where the authors describe concerted ligand transitions and binding site hydration changes to be the main source of the decreased efficiency of the free energy estimation for this protein–ligand system.

**Figure 8:**
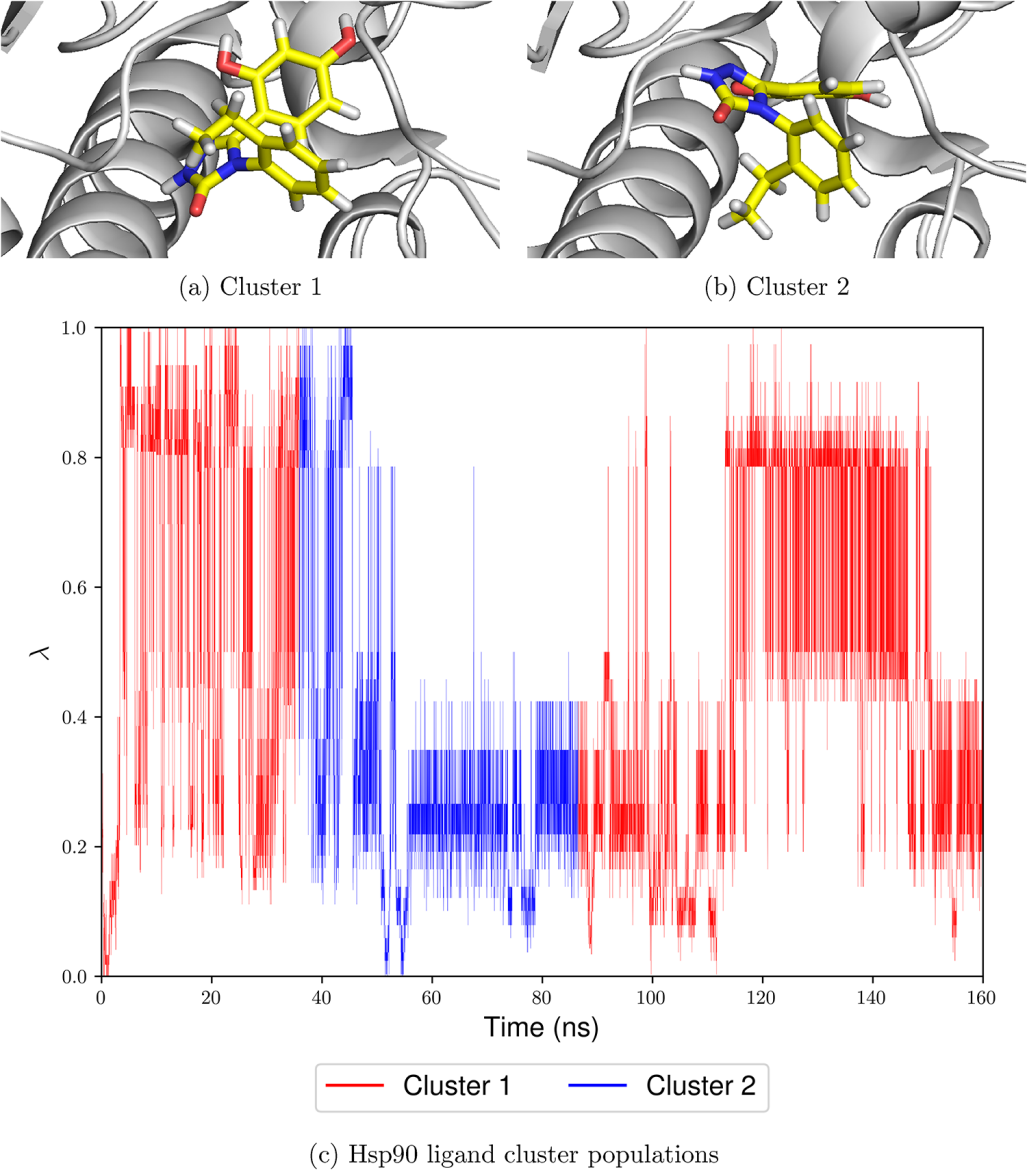
Two ligand common core clusters (a and b) and their transitions in λ space over time (c) during the enhanced outlier simulation in Figure 7d.

In contrast to the previous test cases, not much mobility in the torsional ligand degrees of freedom is observed during the unenhanced protocol, with only the ethyl group torsion (Figure 7a) exhibiting transitions with implied time scales of 0.01 ± 0.00 ns and 0.41 ± 0.00 ns for the unenhanced and the enhanced FAST/MBAR protocols, respectively. Although the two conformations of this torsion (Figure 9a,b) have significantly different median *Δ*Δ*G* ^⊖^ values of 0.03 and 0.34 kcal/mol, respectively (Figure 9c; Kruskal–Wallis *p*-value <0.001), much of the variance in the free energy results is still unexplained by this transition and likely related to various nonbonded interactions between the ligand and the solvated protein.^56–58^ While the enhanced protocol also promotes the rotation of the ethylphenyl ring (Figure 7a) and results in observable transitions with an implied time scale of 0.56 ± 0.00 ns, no reliable cluster-based free energy analysis can be performed in this case due to the low populations of resulting conformers at λ = 1. These low populations show that the extra sampling provided by the enhanced protocol does not significantly improve the quality of the estimated *Δ*Δ*G* ^⊖^ values over the unenhanced FAST/MBAR protocol for this system.

**Figure 9:**
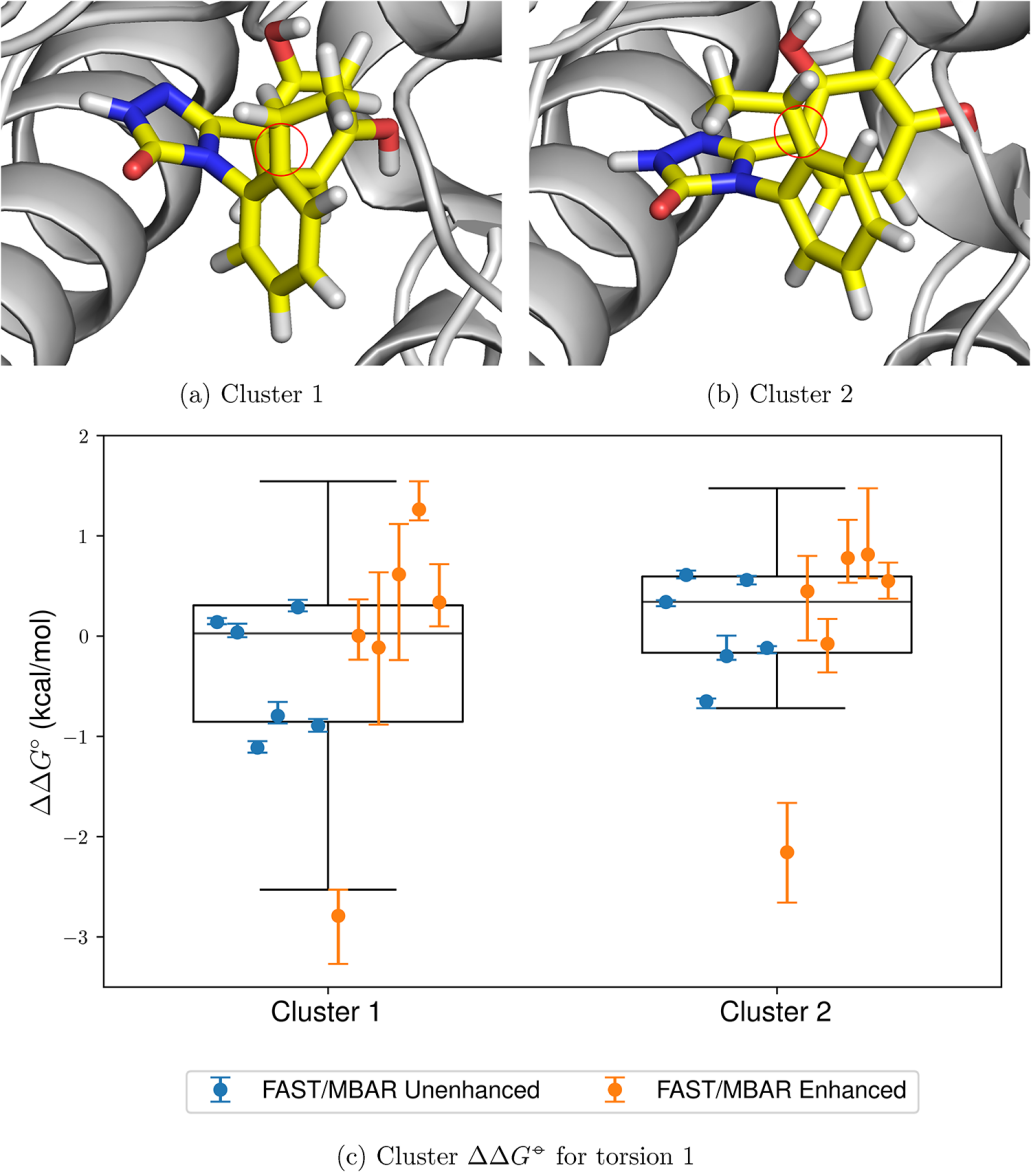
Two different clusters corresponding to torsion 1 (circled in red) in the Hsp90 ligand (a and b) and their respective *Δ*Δ*G* ^⊖^ values obtained from both FAST/MBAR protocols (c). The gray lines represent the median of the total data set, the boxes include the interquartile range, while the whiskers extend to the 5th and 95th percentiles. Each data point represents the median bootstrapped value for each replicate, and the error bars extend between the minimum and maximum bootstrapped values.

Despite the observed decrease in the sampling efficiency of FAST/MBAR compared to the previous test cases, the alchemical protocols are of similar quality to FXa and thrombin, with the unenhanced FAST/MBAR protocol containing 5 ± 1 λ values in total, compared to 7 ± 1 λ values output by the AASMC routine. Similarly, the enhanced FAST/MBAR protocol reduces the λ values from 25 ± 1 to 20 ± 3, behavior also comparable to the previous test cases.

The observed round-trip rate per nanosecond is 8.25 ± 1.63 for the unenhanced protocol, compared to an average of 0.15 ± 0.12 for the enhanced (Kruskal–Wallis *p* = 0.004) (Table 1). These values are consistent with τ̂_decorr_: 1.65 ± 0.22 ps and 12.54 ± 11.17 ps for the unenhanced and enhanced protocol, respectively (Kruskal–Wallis *p* = 0.004) (Table 2). While the added extra sampling significantly decreases the sampling efficiency, these results are still consistent with the previous test cases. A more interesting difference is the lower efficiency of the unenhanced protocol compared to the previous test cases. Although this difference can be partially explained by the slightly higher complexity of the alchemical perturbation compared to the previous test cases, it is also likely influenced by the kinetic trapping in λ space discussed above. Nevertheless, the $\frac{N_{1}}{N_{0}}$ ratios are comparable to the previous systems with values of 0.73 ± 1.42 and 1.13 ± 3.74 for the unenhanced and enhanced FAST/MBAR protocols, respectively (Kruskal–Wallis *p* = 0.57) (Table 3).

### 4.4 Protein Tyrosine Phosphatase 1B (PTP1B)

The final test case is taken from Suruzhon et al.,^6^ where it was demonstrated that significantly different *Δ*Δ*G* ^⊖^ values can be observed after an AFE calculation with a short (100 ps) or a long (20 ns) equilibration protocol. The perturbation of choice (PTP1B, pair 2, PDB ID: 2QBP^59^), shown in Figure 10b,c, exhibits large median differences between both equilibration protocols (larger than 2 kcal/mol)^6^ (Figure 10d,e). To investigate the sensitivity of FAST/MBAR to the equilibration length, two groups of simulations were run: a sextuplicate run starting from the unequilibrated structure and a separate simulation starting from each of the three equilibrated structures generated in Suruzhon et al.,^6^ with two replicates per structure. In both scenarios, the enhanced FAST/MBAR protocol was designed to improve the sampling of the four closest torsions to the perturbed groups. To achieve this, the larger part of the ligand was alchemically decoupled from the rest of the system at λ = 0 (Figure 10a).

**Figure 10:**
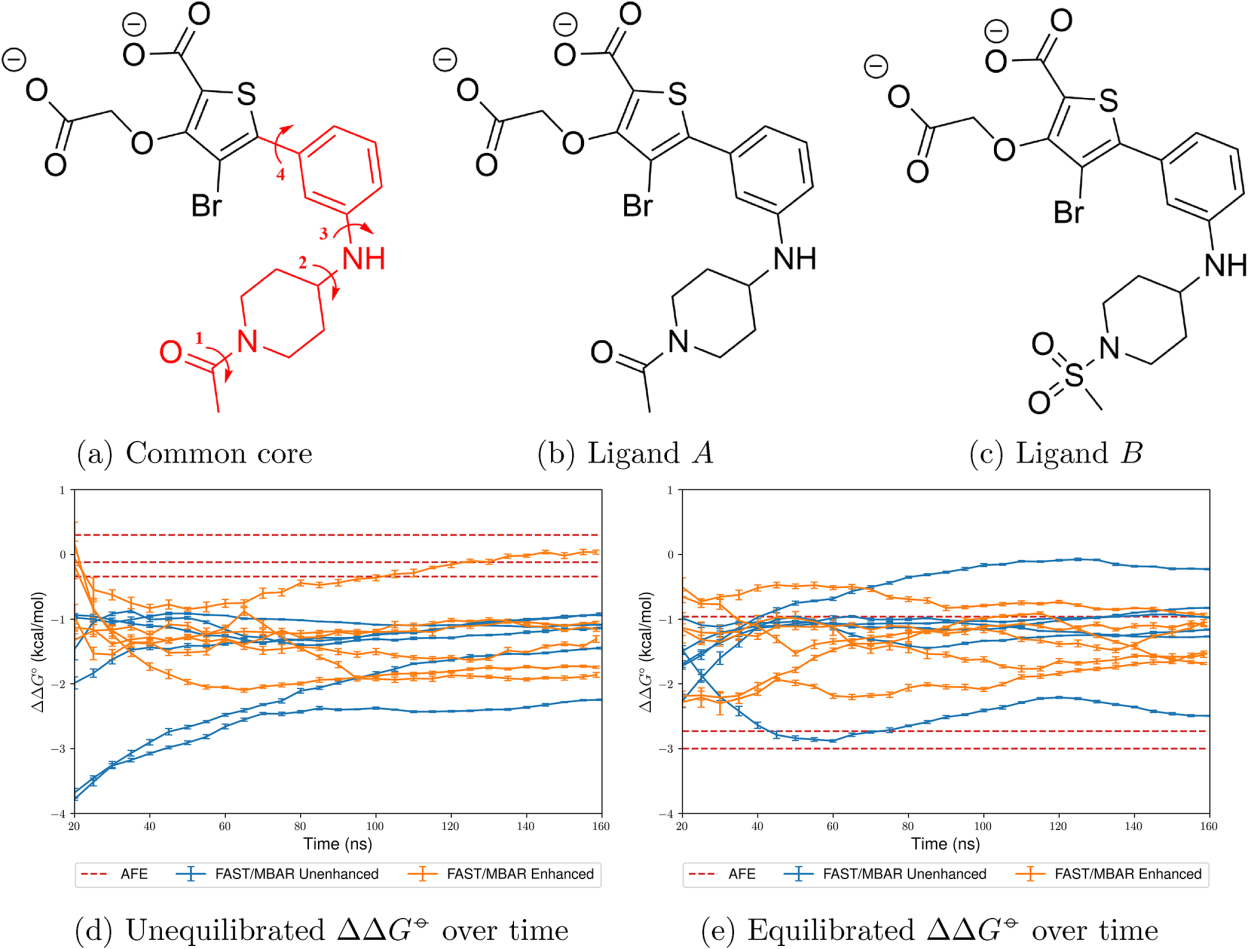
Common core of the PTP1B ligand with dummy atoms and bonds in red (a), the two ligands constituting the relative free energy perturbation (b and c) and the bootstrapped *Δ*Δ*G* ^⊖^ estimates corresponding to both FAST/MBAR protocols over time for the unequilibrated (d) and the equilibrated (e) structures. In (d) to (e), the median *Δ*Δ*G* ^⊖^ for each replicate is plotted over time with associated error bars determined by the bootstrapped MAD, while *Δ*Δ*G* ^⊖^ determined by each AFE replicate is shown as a separate red line.

Interestingly, after 20 ns, the FAST/MBAR protocol without enhanced sampling results in less variable free energy values for the equilibrated structures, compared to the unequilibrated ones, with a MAD of 0.29 and 1.01 kcal/mol, respectively (Figure 10d,e). Furthermore, this difference in MAD is significant at the 1% level with a Kruskal–Wallis *p*-value of 0.01 when comparing the two respective populations of inter-replicate absolute deviations. This is a surprising result, since the extra decorrelation provided by the additional equilibration is expected to introduce uncertainty into the free energy estimates instead of reducing it. Nevertheless, comparison between the free energy results obtained from the unequilibrated and the equilibrated protocol yields a high Kruskal–Wallis *p*-value of 1.0, due to the large inter-replicate MAD, meaning that the two sets of free energy values are statistically indistinguishable. After 160 ns, both types of unenhanced FAST/MBAR simulations result in comparable inter-replicate MADs of 0.31 kcal/mol (unequilibrated) and 0.48 kcal/mol (equilibrated), respectively (Kruskal–Wallis *p* = 0.17), as well as comparable free energy results (Kruskal–Wallis *p* = 0.75), again indicating the statistical indistinguishability of both sets of simulations.

The enhanced FAST/MBAR protocol results in the same variability for the unequilibrated and equilibrated structures after 20 ns of simulation (0.56 kcal/mol MAD). After 160 ns, the variability decreases for both protocols, with inter-replicate MADs of 0.46 and 0.28 kcal/mol for the unequilibrated and the equilibrated simulations, respectively (Figure 10d,e), and a Kruskal–Wallis *p*-value of 0.05. This suggests that the FAST/MBAR protocol goes some way to achieving a similar level of decorrelation as that provided by extensive equilibration.

As with the Hsp90 test case, there is an outlier which is kinetically trapped in λ space, thereby reducing the reliability of the estimated free energies (Figure 11). The largest outlier in this case is one of the unequilibrated enhanced simulations with the final *Δ*Δ*G* ^⊖^ value of 0.03 kcal/mol. The kinetic barriers are correlated with three main common core conformers (Figure 11a–c), whose transitions are again observed at highly decoupled states. In this case, however, the conformers are significantly sampled at λ = 0.5, which corresponds to the fully coupled amide derivative. Therefore, these conformers are much more energetically favorable for one of the ligands, thereby creating a bottleneck in the part of the protocol responsible for the relative free energy calculation (0.5 ≤ λ ≤ 1.0).

**Figure 11:**
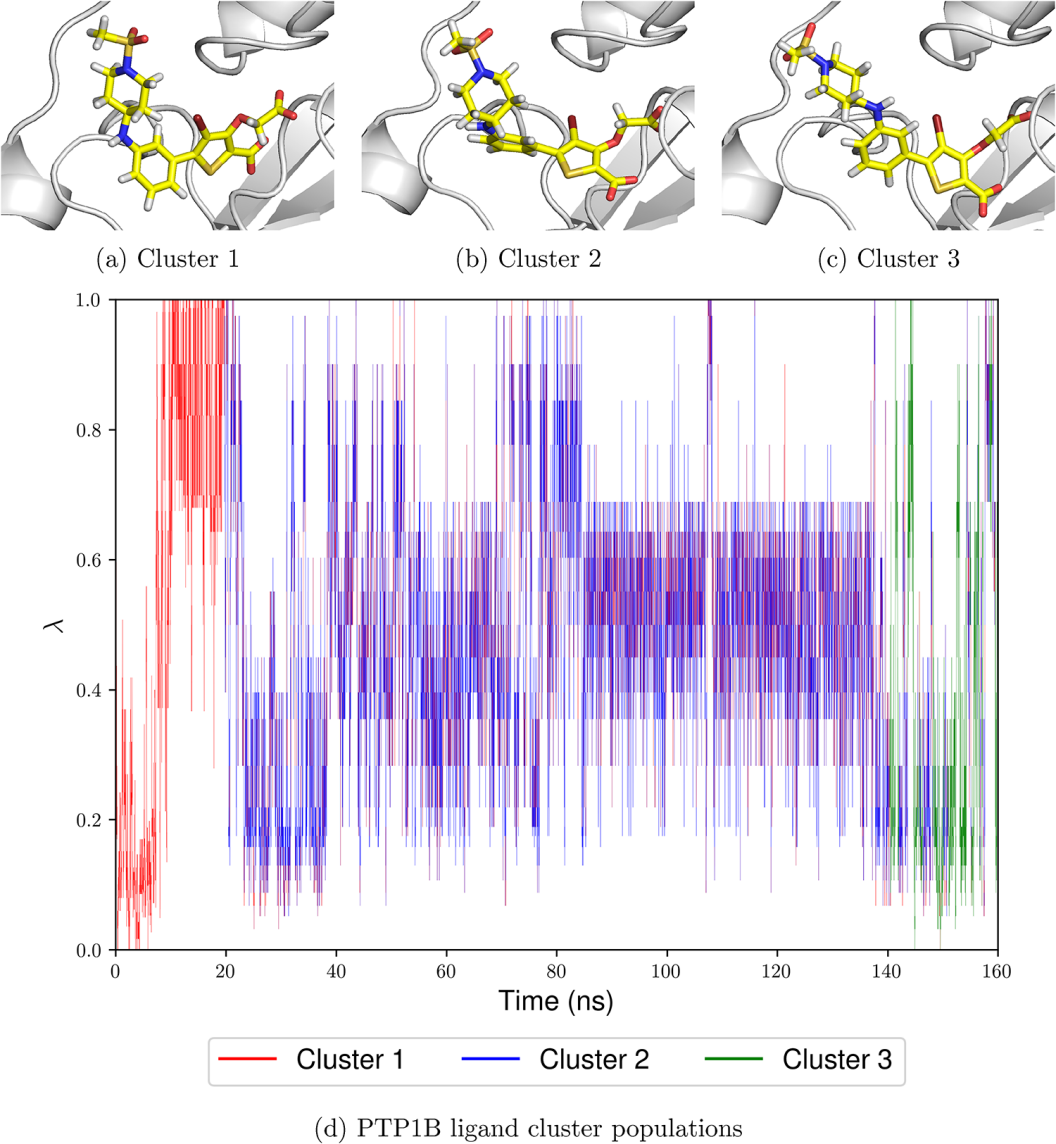
Three PTP1B ligand common core clusters (a–c) and their transitions in λ space over time (d) during the unequilibrated, enhanced outlier simulation in Figure 10d.

Analysis of the free energy values as a function of the torsional degrees of freedom is difficult for this highly flexible ligand (Figure 10a), due to the large number of possible conformers, leading to a large uncertainty in the estimated *Δ*Δ*G* ^⊖^ values. The degree of freedom showing the highest per-cluster free energy convergence is torsion 4 (Figure 10a), which has three associated conformations (Figure 12a–c). These three clusters have significantly different respective *Δ*Δ*G* ^⊖^ values of 0.02, −0.22 and −0.82 kcal/mol for the unequilibrated protocol (Kruskal–Wallis *p*-value ≪ 0.001), and 0.09, −0.34 and −0.43 kcal/mol for the equilibrated protocol with a Kruskal–Wallis *p*-value of 0.01 (Figure 12d,e). There is large uncertainty in all per-cluster *Δ*Δ*G* ^⊖^ values, primarily caused by undersampling of cluster 3 and increased sampling of clusters 1 and 2, although instances of undersampling can be seen for all clusters. Nevertheless, comparison between the median *Δ*Δ*G* ^⊖^ values of cluster 1 and cluster 2 without sampling enhancement yields differences of 0.19 and 0.62 kcal/mol for the unequilibrated and equilibrated FAST/MBAR protocols, respectively (Kruskal–Wallis *p*-value ≪ 0.001 for the comparison of the clusters across both protocols), showing that this degree of freedom can have a significant effect on the obtained free energy values. The slowest and most significantly enhanced degree of freedom, however, is torsion 3, with an implied time scale of 0.71 ± 0.06 ns after enhancement, compared to 14.66 ± 3.84 ns without enhancement (unequilibrated protocol), as determined by the MSM analysis. These results again show that even high kinetic barriers with implied time scales on the order of 10 ns can be readily explored by the unenhanced protocol over the range of 160 ns, thereby demonstrating the robustness of FAST/MBAR toward the initial conformer used compared to standard AFE calculation methods.

**Figure 12:**
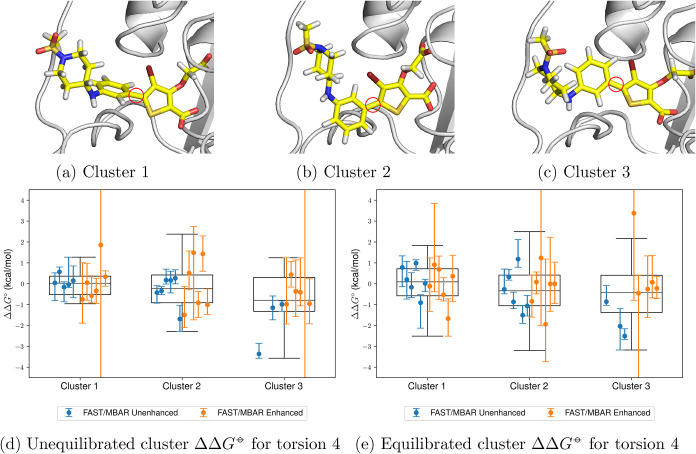
Three different clusters corresponding to torsion 4 (circled in red) in the PTP1B ligand (a–c) and their respective *Δ*Δ*G* ^⊖^ values obtained from both FAST/MBAR protocols for the unequilibrated (d) and the equilibrated (e) structures. The gray lines represent the median of the total data set, the boxes include the interquartile range, while the whiskers extend to the 5th and 95th percentiles. Each data point represents the median bootstrapped value for each replicate, and the error bars extend between the minimum and maximum bootstrapped values.

This test case is significantly more challenging than the previous test cases, as evidenced by the high number of required λ windows. Their initial number generated by the initial AASMC procedure can be as high as 34 for the enhanced protocol and 13 for the unenhanced protocol. This large number of intermediates is caused by a combination of a challenging decoupling procedure of a large part of the ligand and a relative perturbation involving transformations in five of the bonded terms, arising from the atom type change of the sulfonamide sulfur and nitrogen atoms. Although the FAST protocol optimization procedure reduces the average number of λ windows to 8–11 for the unenhanced protocol in both the equilibrated and unequilibrated simulations, a more peculiar behavior is observed for the enhanced protocol. In this case, the λ values of the simulations starting from the unequilibrated structures are reduced to 25 ± 6, while all of the equilibrated simulations retain the same protocol as the initial AASMC procedure throughout the simulations, resulting in longer protocols (33 ± 1 λ values). While this observation is clearly caused by the inability of the optimizer to find a more favorable protocol and it can be partially explained by the high complexity of the protocols, it is not obvious why the optimization procedure is successful for most of the unequilibrated simulations. A possible explanation is that the unequilibrated structures result in more unfavorable AASMC protocols, which makes finding a new minimum easier for the FAST/MBAR protocol optimization procedure.

Similarly to the previous test cases, the increased complexity of the protocols is reflected by the substantially reduced round-trip rate per nanosecond: 0.07 ± 0.02 and 1.58 ± 0.46 for the enhanced and unenhanced FAST/MBAR protocols, respectively (Kruskal–Wallis *p* ≪ 0.001) (Table 1). The effective decorrelation time remains comparable to the previous test cases, although it differs significantly between protocols, with 8.64 ± 3.91 ps for the protocol with extra sampling and 2.91 ± 1.08 ps for the unenhanced protocol (Kruskal–Wallis *p* ≪ 0.001) (Table 2). In all cases, the values are comparable between the equilibrated and unequilibrated simulations. As in the previous systems, the $\frac{N_{1}}{N_{0}}$ ratios are generally satisfactory for both protocols, with values of 0.71 ± 1.51 for the unenhanced protocol and 1.29 ± 3.28 for the enhanced protocol, the latter being primarily due to a few outlier runs (Kruskal–Wallis *p* = 0.13) (Table 3).

## 5 Discussion

The above test cases demonstrate that the FAST framework is not only capable of enhancing the sampling of specific degrees of freedom, as shown in ref 37, but can also be readily employed in the context of binding free energy calculations with or without extra targeted sampling. This makes FAST/MBAR a robust, flexible, reproducible, and fully automatable alternative to standard free energy calculation methods.

Comparing the behavior between FAST/MBAR free energy calculations with and without enhanced sampling is of particular interest, since it can directly show the impact of enhanced sampling on the efficiency and quality of the free energy calculations. The FXa and thrombin results show a somewhat expected trend: at relatively short time scales (∼20 ns), the free energy values obtained from the FAST simulations without extra sampling are similar to the short AFE calculations, while at longer time scales, they tend toward the values predicted by the enhanced protocol. This observation is readily explained by the fact that at longer time scales even higher torsional kinetic barriers can be surmounted with regular MD.

In contrast to FXa and thrombin, the Hsp90 results show large variance in the obtained free energy values and, although this variance decreases over time, it remains substantially higher than for the other test cases. Normally this would be an unexpected result, since the apparent complexity of the ligand is comparable to the other better-behaved test cases. However, it is well-known that Hsp90 can be a problematic system for molecular simulations, due to its high mobility and its closed binding site, which makes binding site ligand and water sampling significantly more challenging.^56,60,61^ Indeed, it is shown here that the variability in free energy values is caused by slow transitions of the common core at highly decoupled λ values—an observation which is reminiscent of the kinetically trapped TGF-β system considered in refs 37,45 . In addition, the extra conformers provided by the enhanced FAST/MBAR are not very stable at the fully coupled ligand states, resulting in increased effective correlation and further reduced efficiency. These two manifestations of kinetic trapping in λ space make Hsp90 a much more challenging system to study than FXa and thrombin.

The PTP1B test case shows that even highly flexible large ligands can be handled by FAST/MBAR. However, turning off the nonbonded interactions of a large part of the ligand can lead to the same problem of slow common core transitions at more decoupled states, as observed in Hsp90 and TGF-β.^29,37^ Despite this challenge, the robustness of FAST/MBAR with respect to decorrelation from the initial coordinates was shown to be very high and FAST/MBAR provides significant improvement over traditional AFE calculations.

It is therefore clear that both FAST/MBAR protocols have advantages and disadvantages and the superiority of one of them over the other is not obvious. On one hand, FAST/MBAR without extra sampling results in a large number of round trips, which in turn yields a higher amount of sampling at the states of interest and therefore higher confidence in the calculated free energies. On the other hand, extra sampling of the relevant degrees of freedom can provide a higher consistency of the free energy values over time, as well as output a better converged distribution of conformers, if these are of interest. Indeed, the above results show that the free energy differences associated with a particular conformer can differ by nearly 1 kcal/mol, meaning that sampling different conformers as efficiently as possible is crucial for minimizing the initial-structure bias of the free energy calculation. It is equally as important that the enhanced region is carefully selected, however, as large regions can significantly decrease the efficiency of the algorithm by creating kinetic barriers in λ space. Similarly, enhancing the sampling of an unimportant degree of freedom will simply result in increased computational expense to no benefit. As this information is usually not known in advance, it follows that enhanced sampling of the ligand degrees of freedom should be done sparingly on only several key degrees of freedom, such as torsions that are close to the perturbed group, or ones that are expected to have very high kinetic barriers. The sampling of the other degrees of freedom can then be handled by the MD integrator, especially since the single-trajectory nature of FAST provides maximum decorrelation from the initial coordinates.

The above results also demonstrate the utility of the bond rescaling procedure in handling perturbations of harmonic bond potentials. For example, the addition of an extra methyl group required only 1–2 intermediate states for both FXa and thrombin, while Hsp90 needed 3–5 intermediate λ windows for the addition of an ethyl group. In cases where the bond rescaling procedure could not be used to handle all bonds, the number of required intermediates can be significantly higher—e.g., between 6 and 9 for PTP1B. In any case, however, the number of these intermediates is always optimal, as determined by the FAST protocol optimization procedure. In this way, FAST maximizes the mobility in λ space, as well as the amount of sampling at the endstates. The only case where the optimization procedure failed to provide improvement was for the very long explicitly enhanced PTP1B protocols, which had over 30 starting intermediate λ values predicted by the initial AASMC routine. Alternative optimization algorithms may need to be considered in the future for such challenging cases.

The advantages of FAST/MBAR over conventional fixed-protocol AFE calculation methods are clear: the completely automated procedure which does not depend on a provided alchemical protocol achieves good sampling efficiency, while the single-trajectory nature of the method provides maximum long-time scale decorrelation and improved robustness to the choice of initial coordinates. Although the use of an optimal λ protocol does not, by itself, guarantee improved accuracy in the final free energy estimates, it is expected to minimize the uncertainty in the results.^62–64^ In contrast, there is always a trade-off between the number of λ values and the simulation time per intermediate in regular AFE calculations—too few intermediates will lead to poor free energy estimation due to insufficient phase space overlap, while too many will result in decreased computational time per λ window, and therefore increased correlation and bias to their initial coordinates.

While FAST/MBAR eliminates the most significant weakness of traditional AFE methods, it is important to note that this comes at a price. First, FAST/MBAR does not result in equal sampling over λ space at a finite number of samples, meaning that the amount of time spent in a particular λ state is not known *a priori*. This also means that FAST/MBAR is particularly sensitive to kinetic barriers in λ space or slow events which can shift the free energy profile over time. Finally, there is an extra computational cost associated with the protocol optimization procedure, as well as the need to estimate free energy values during the simulation.

Arguably the most significant of these weaknesses is the sensitivity toward kinetic barriers in λ space, since addressing it would significantly improve the sampling homogeneity in λ space, as the free energy values start converging. Similarly, the extra computational cost can be either tackled by performing protocol and free energy updates less frequently, or by using an implementation which performs these in the background on the CPU (central processing unit), while the simulation is running on the GPU (graphics processing unit). Since GPUs are becoming increasingly more widely used in molecular simulations,^65–68^ this effectively means that the extra CPU overhead posed by FAST could be made practically negligible with a sufficiently optimized implementation.

Future work should therefore focus on improving the robustness of the algorithm toward systems exhibiting high kinetic barriers in λ space. Nevertheless, such barriers are not expected for relatively simple alchemical perturbations, meaning that FAST/MBAR is already a suitable method for performing automated and robust free energy calculations. In addition to its adaptive nature, FAST/MBAR is also extremely general and can be readily extended to more sophisticated applications, such as combining free energy calculations with several types of enhanced sampling over different amino acid protonation states. This is another promising avenue for future research which will increase the reliability of free energy calculations performed by FAST/MBAR further.

## 6 Conclusion

In this work the FAST (fully adaptive simulated tempering) method presented in ref 37 was extended to the context of relative free energy calculations. The resulting method, FAST/MBAR, is highly efficient and enables the automation of alchemical free energy calculations in a robust way, as demonstrated on four different protein–ligand systems.

It was shown that combining FAST/MBAR with enhanced sampling can decrease the short-time scale bias of the free energy values toward the initial structure at the cost of reduced mobility in λ space. On the other hand, free energy protocols without extra sampling retain this bias at longer time scales. In both cases, however, many ligand rare events can be readily explored due to the single-trajectory nature of the method. In conjunction with its automated nature, this makes FAST/MBAR a reliable alternative to standard AFE calculation methods.

The above results also illustrate how large perturbations and/or orthogonal slow motions can have a detrimental effect on the efficiency of FAST/MBAR in λ space, as well as the convergence of the free energies. Although this consideration is not exclusive to FAST/MBAR, and is instead applicable to all alchemical/tempering methods, it is still an important topic to address in future work. In addition, the flexibility of the FAST framework can be used for enhancing the sampling of many different parts of the system over different protonation states. Combining this with free energy calculations is another area of interest for further research.

## Supplementary Material







## Data Availability

The data supporting this study are available in the Supporting Information and from the Zenodo repository at DOI: 10.5281/zenodo.20795812. Source code for OpenMMSLICER is available at https://github.com/essex-lab/OpenMMSLICER.

## Notes

Relevant raw data (XLSX)
Python scripts used to perform the study using OpenMMSLICER 3.0.0 (ZIP)
Derivation of the bond rescaling Jacobian and a comparison of the free energy results with available experimental data (PDF)
