# Supplementary material for: Fully Automatable Relative Binding Free Energy Calculations with Enhanced Sampling Using FAST/MBAR
Source: J Chem Theory Comput. 2026 Jul 23;22(15):7993–8012. doi: 10.1021/acs.jctc.6c00963 (PMC13472099; doi:10.1021/acs.jctc.6c00963)
Supplement: Supplementary file 1 [file ct6c00963_si_003.pdf]

Supporting Information for Fully Automatable Relative Binding Free Energy Calculations with Enhanced Sampling using FAST/MBAR

Miroslav Suruzhon,[†] Justina Ratkeviciute,[†] Khaled Abdel-Maksoud,[†] Anna Cavalleri,[†] Michael S. Bodnarchuk,[‡] Antonella Ciancetta,[¶,§] Ian D. Wall,[‖] and Jonathan W. Essex[*,†]

†*School of Chemistry and Chemical Engineering, University of Southampton, Highfield, Southampton SO17 1BJ, United Kingdom*

‡*Computational Chemistry, R&D Oncology, AstraZeneca, Cambridge CB4 0WG, United Kingdom*

¶*Sygnature Discovery Limited, BioCity, Pennyfoot St, Nottingham NG1 1GR, United Kingdom*

§*Department of Chemical, Pharmaceutical and Agricultural Sciences (DOCPAS), Via Fossato di Mortara 17/19, 44121, Ferrara, University of Ferrara*

‖*GSK Medicines Research Centre, Gunnels Wood Road, Stevenage SG1 2NY, United Kingdom*

E-mail: J.W.Essex@soton.ac.uk

1 Derivation of the Bond Rescaling Jacobian

The elements of the Jacobian matrix $\mathbf{J}_{\vec{T}}(\vec{x})$ corresponding to a transformation $\vec{T}$ are defined as follows:

$$J_{ij,\vec{T}}(\vec{x}) = \frac{\partial T_i(\vec{x})}{\partial x_j} \tag{1}$$

A bond rescaling move between two atoms with coordinates $\vec{r}_0$ and $\vec{r}_1$ by a scaling factor s can be considered as a translation of $\vec{r}_1$ and all atoms bonded to it $\vec{r}_{movable}$ by $(s-1)(\vec{r}_1-\vec{r}_0)$:

$$\vec{T}(\vec{r}_{movable}) = \vec{r}_{movable} + (s-1)(\vec{r}_1 - \vec{r}_0) \tag{2}$$

To calculate the determinant $|\mathbf{J}_{\vec{T}}(\vec{x})|$, we first note that $J_{ij,\vec{T}}(\vec{x}) = 0$ for all row elements $i \neq j$ corresponding to the stationary atoms, since their coordinates remain unchanged. Afterwards, we use the following well-known identity for the determinant of a block matrix $\mathbf{M} = \left(\begin{smallmatrix}\mathbf{A} & \mathbf{B}\\ \mathbf{C} & \mathbf{D}\end{smallmatrix}\right)$:

$$|\mathbf{M}| = |\mathbf{A} - \mathbf{B}\mathbf{D}^{-1}\mathbf{C}||\mathbf{D}| \tag{3}$$

If either $\mathbf{B}$ or $\mathbf{C}$ have only zero elements, the determinant reduces to:

$$|\mathbf{M}| = |\mathbf{A}||\mathbf{D}| \tag{4}$$

Therefore, all off-diagonal column elements corresponding to the stationary atoms do not contribute to the Jacobian determinant, even if they are not zero. This argument can be then iteratively applied to conclude that in our setting only the diagonal elements of each atom contribute to the determinant. For the transformation shown in Equation (2), all diagonal terms are unity except for the three coordinates corresponding to $\vec{r}_1$, where each element is equal to s. Therefore, the Jacobian determinant for a bond rescaling transformation by a scaling factor s is:

$$|\mathbf{J}_{\vec{T}}(\vec{x})| = s^3 \tag{5}$$

This procedure can be straightforwardly generalised to multiple concurrent reversible

bond rescaling transformations with scaling factors $s_1, ..., s_N$, in which case the Jacobian determinant is:

$$|\mathbf{J}_{\vec{T}}(\vec{x})| = \prod_{i=1}^{N} s_i^3 \tag{6}$$

2 Comparison to Available Experimental Data

Table 1: Comparison of calculated and available experimental $\Delta\Delta G^{\circ}$ values. All values are in kcal/mol and reported as median (MAD).

	System				
Method	FXa	Thrombin	HSP90	PTP1B, uneq.	PTP1B, eq.
FAST w/o ES	-0.11 (0.15)	0.17 (0.44)	-0.10 (0.45)	-1.11 (0.31)	-1.07 (0.48)
FAST with ES	-0.17 (0.12)	0.12 (0.07)	0.22 (0.79)	-1.23 (0.46)	-1.30 (0.28)
AFE	-0.38 (0.01)	1.12 (0.06)	-0.21 (0.10)	-0.12 (0.21)	-2.73 (0.68)
Experimental	-1.01[1]	—	1.19[2]	-0.87[3]	

References

(1) Matter, H.; Defossa, E.; Heinelt, U.; Blohm, P.-M.; Schneider, D.; Müller, A.; Herok, S.; Schreuder, H.; Liesum, A.; Brachvogel, V.; Lönze, P.; Walser, A.; Al-Obeidi, F.; Wildgoose, P. Design and Quantitative Structure-Activity Relationship of 3-Amidinobenzyl-1H-Indole-2-Carboxamides as Potent, Nonchiral, and Selective Inhibitors of Blood Coagulation Factor Xa. *J. Med. Chem.* **2002**, *45*, 2749–2769.

(2) Amaral, M.; Kokh, D. B.; Bomke, J.; Wegener, A.; Buchstaller, H. P.; Eggenweiler, H. M.; Matias, P.; Sirrenberg, C.; Wade, R. C.; Frech, M. Protein Conformational Flexibility Modulates Kinetics and Thermodynamics of Drug Binding. *Nat. Commun.* **2017**, *8*, 2276.

(3) Wilson, D. P.; Wan, Z.-K.; Xu, W.-X.; Kirincich, S. J.; Follows, B. C.; Joseph-McCarthy, D.; Foreman, K.; Moretto, A.; Wu, J.; Zhu, M.; Binnun, E.; Zhang, Y.-L.; Tam, M.; Erbe, D. V.; Tobin, J.; Xu, X.; Leung, L.; Shilling, A.; Tam, S. Y.; Mansour, T. S.; Lee, J. Structure-Based Optimization of Protein Tyrosine Phosphatase 1B Inhibitors: From the Active Site to the Second Phosphotyrosine Binding Site. *J. Med. Chem.* **2007**, *50*, 4681–4698.
